# Combinational Analysis of Metabolomic and O-GlcNAcylation Omics Reveals the HBP Metabolic Regulation of Chemoresistance via GFPT1/NR3C1 O-GlcNAcylation/GPX4 Axis

**DOI:** 10.34133/research.0809

**Published:** 2025-07-30

**Authors:** Xiang Zhou, Chunlin Zhang, Li Li, Zhenwei Feng, Xuesong Bai, Yuhua Mei, Weiyang He, Xin Gou, Xinyuan Li

**Affiliations:** ^1^ Department of Urology, The First Affiliated Hospital of Chongqing Medical University, Chongqing, China.; ^2^ Chongqing Key Laboratory of Molecular Oncology and Epigenetics, Chongqing, China.

## Abstract

Dysregulation of ferroptosis is linked to chemoresistance, and reprogramming of glucose metabolism is involved in this progress. However, the underlying mechanisms remain obscure. Herein, using metabolic profiling, we find that hexosamine biosynthetic pathway (HBP) metabolism and the byproduct, UDP-GlcNAc, are substantially up-regulated in chemoresistant tumor tissues and cells. UDP-GlcNAc-derived O-GlcNAcylation levels increase with the decreased ferroptosis and chemosensitivity in cancer cells. Knockout of the rate-limiting enzyme GFPT1 in HBP metabolism inhibits O-GlcNAcylation, induces ferroptosis, and mitigates chemoresistance of orthotopic bladder cancer in *Gfpt1^−/−^* mice. The global O-GlcNAcylation omics mapped the O-GlcNAcylated sites and proteins in resistant and nonresistant tumor cells, showing that NR3C1 is highly O-GlcNAcylated at Thr^299^ in response to chemotherapy. The chromatin immunoprecipitation sequencing delineates that NR3C1 O-GlcNAcylation at Thr^299^ prominently enhances transcriptional activity of GPX4 by facilitating the binding of NR3C1 on GPX4 promoter, inhibiting ferroptosis. Higher O-GlcNAcylation of NR3C1 improves protein stability and reduces proteasome-dependent degradation by suppressing ubiquitination. Inhibition of NR3C1 O-GlcNAcylation via Thr^299^ mutant or knockout of NR3C1 facilitates ferroptosis and improves chemosensitivity of resistant cancer cells in vitro and in vivo. In addition, we propose a novel predicting model for chemoresistance based on the GFPT1 and NR3C1 levels in pre-chemotherapy biopsy tissues through a training set and a validation set. These findings exemplify how metabolic and epigenetic reprogramming regulates ferroptosis via the GFPT1/NR3C1/GPX4 axis, and implicate NR3C1 O-GlcNAcylation as a potential target for reversing chemoresistance.

## Introduction

Bladder cancer (BCa) causes an age-standardized mortality rate of 3.3% for men versus 0.86% for women worldwide [1], of which muscle invasive bladder cancer (MIBC) is more aggressive with a 5-year survival rate of less than 15% [2,3]. Currently, neoadjuvant chemotherapy consisting of gemcitabine and cisplatin (GC) confers a prominent advantage for improving the survival rate of MIBC patients and even achieving bladder preservation [4]; however, the long-term efficacy is limited because of a high incidence of developing resistance, resulting in increased recurrence and mortality [5]. Therefore, it is imperative to unravel resistant mechanisms and decipher novel therapeutic targets.

Ferroptosis is a unique form of regulated necrosis, triggered by the iron-dependent lipid peroxidation [6,7]. There is a growing appreciation of the role of ferroptosis on provoking chemotherapy resistance in a variety of tumors [8]. Mounting preclinical evidence indicates that ferroptosis might be a promising therapeutic target to mitigate chemotherapy resistance [9,10]. In pancreatic tumor, cancer-associated fibroblast (CAF)-derived long-chain fatty acid–CoA ligase 4 (ACSL4)-targeting miRNAs suppress ferroptosis and induce gemcitabine resistance [10]. CAF-derived exosomal microRNA-522 attenuates ferroptosis by down-regulating Arachidonate 15-lipoxygenase (ALOX15), thus provoking acquired cisplatin resistance in gastric cancer [11]. More importantly, the susceptibility of different cancer cells to ferroptosis is prominently diverse, and the role of ferroptosis on GC resistance in BCa has remained largely unexplored. Therefore, a better understanding of the intrinsic relevance between ferroptosis and GC resistance will benefit the discovery of new therapeutic targets for BCa.

Metabolic reprogramming is tightly connected to ferroptosis, while the regulatory pathways are varied owing to complex metabolic pattern and obvious heterogeneity of tumor cells [12,13]. In the previous study, we identified that the up-regulation of hexosamine biosynthetic pathway (HBP) induced ferroptosis by BTB domain and CNC homolog 2 (BACH2)-mediated solute carrier family 7 member 11 (SLC7A11) repression [14]. The HBP metabolism is a branch of the aerobic glycolysis for directly synthesizing the nucleotide sugar UDP-GlcNAc, providing substrate for O-linked 𝛽-N-acetylglucosamine (O-GlcNAc) protein modification (O-GlcNAcylation) [15,16]. HBP metabolism-induced O-GlcNAcylation has been exhibited to modulate numerous tumor behaviors, such as angiogenesis and chemotherapy resistance [17]. We found that seryl-TRNA synthetase 1 (SerRS) O-GlcNAcylation in endothelial cells boosts the angiogenesis of BCa [18]. Inflammatory interferon-induced protein with tetratricopeptide repeats 3 (IFIT3) regulates voltage-dependent anion channel 2 (VDAC2) O-GlcNAcylation and promotes the gemcitabine and paclitaxel resistance in pancreatic ductal adenocarcinoma cells [19]. However, the intrinsic connection among HBP metabolism, ferroptosis, and chemoresistance is unclear.

The nuclear receptor subfamily 3 group C member 1 (NR3C1) gene encodes the glucocorticoid receptor binding to glucocorticoid response elements, but NR3C1 can activate gene transcription in a hormone-independent manner. A growing number of evidences indicate the important roles of NR3C1 in cancer proliferation, metastasis, and drug resistance. NR3C1 induces reversible cancer cell dormancy by activating growth factor survival signaling [20]. NR3C1 drove the formation of phase-separation droplets around super enhancers and induced gastric cancer resistance to 5-fluorouracil [21]. The binding between NR3C1 and glucocorticoids facilitated formation of neutrophil extracellular traps and lung cancer metastasis [22]. The transcriptional regulation effects of NR3C1 on tumor progression remain worthy of further investigation.

In the current study, we uncover a novel metabolic and epigenetic mechanism underlying ferroptosis and inducing chemoresistance. Abnormal up-regulation of glutamine–fructose-6-phosphate transaminase 1 (GFPT1) triggers HBP metabolic reprogramming, which facilitates NR3C1 O-GlcNAcylation at threonine 299 (Thr^299^). NR3C1 O-GlcNAcylation further improves glutathione peroxidase 4 (GPX4) transcriptional activity and inhibits ferroptosis by increasing NR3C1 stability and its binding to GPX4 promoter. In addition, we propose a novel predicting model for chemoresistance based on the GFPT1 and NR3C1 expression in pre-chemotherapy biopsy tissues. Taken together, our findings indicate that targeting the GFPT1/NR3C1 O-GlcNAcylation/GPX4 axis might be an effective strategy to overcome chemoresistance.

## Results

### GFPT1 is up-regulated in chemoresistant tumor tissues and cells

**Fig. 1.**
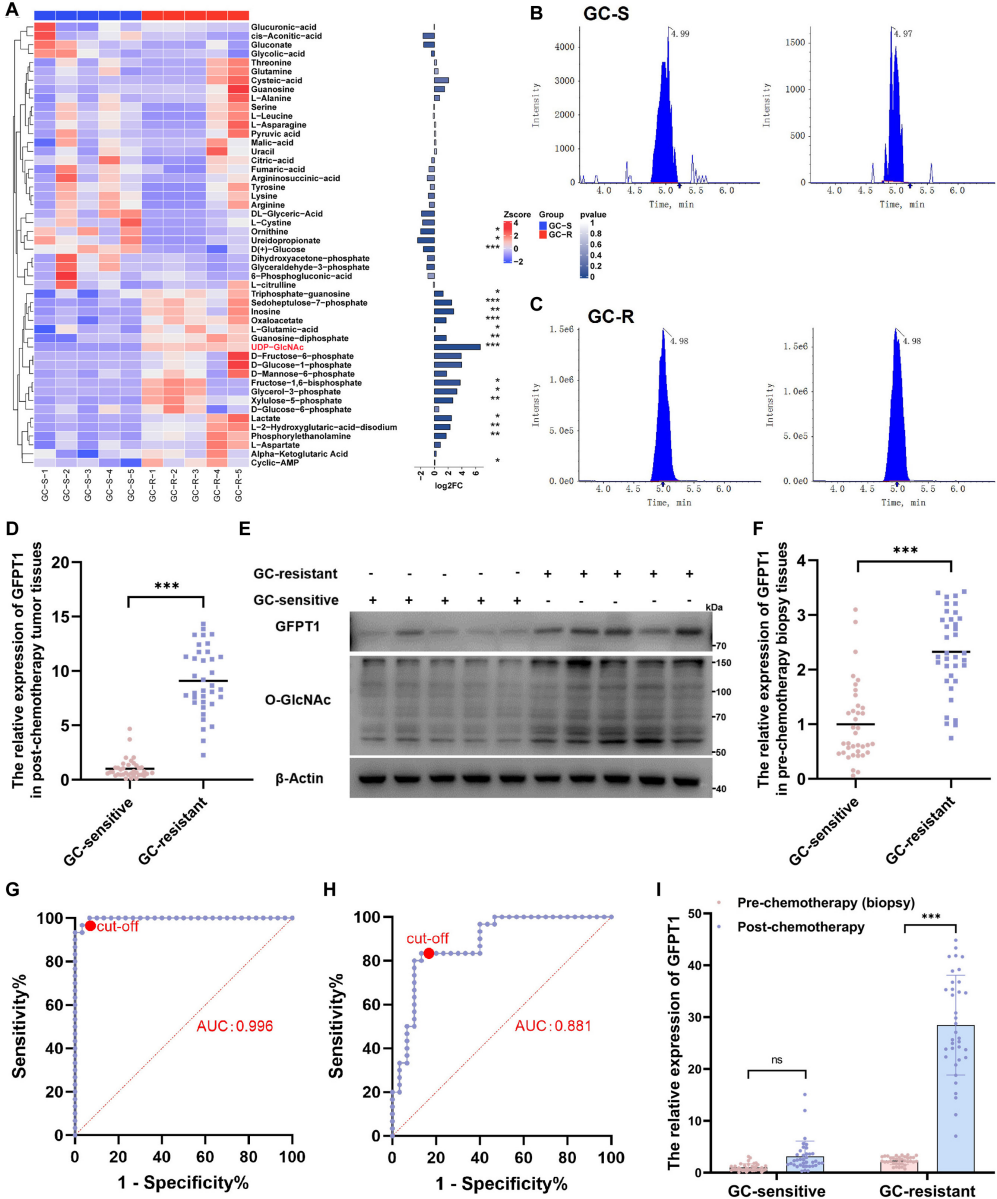
GFPT1 is up-regulated in GC-resistant BCa tissues. (A) Cluster heat map illustrating the differential metabolites in energy metabolism and the fold changes between GC-sensitive patients (GC-S, *n* = 5) and GC-resistant patients (GC-R, *n* = 5). (B and C) UDP-GlcNAc content in GC-sensitive patients (*n* = 5) and GC-resistant patients (*n* = 5). (D) Relative GFPT1 transcriptional expression in post-chemotherapy tumor tissues from GC-sensitive (*n* = 37) and GC-resistant (*n* = 36) BCa patients; normalized according to the GFPT1 levels of GC-sensitive BCa tissues; unpaired 2-tailed Student’s *t* test. (E) Protein levels of GFPT1 and O-GlcNAc in radical cystectomy BCa tissues of GC-sensitive patients and GC-resistant patients. (F) Relative GFPT1 transcriptional expression in pre-chemotherapy biopsy tumor tissues from GC-sensitive (*n* = 37) and GC-resistant (*n* = 36) BCa patients; normalized according to the GFPT1 levels of GC-sensitive biopsy tumor tissues; unpaired 2-tailed Student’s *t* test. The ROC curves of the early prediction model for effectiveness of GC treatment based on the expression levels of GFPT1 in post-chemotherapy (95% CI: 0.9891 to 1.000) (G) and pre-chemotherapy biopsy (95% CI: 0.7937 to 0.9685) (H) BCa tissues. (I) GFPT1 levels in pre-chemotherapy and post-chemotherapy BCa tissues from GC-sensitive patients (*n* = 37) and GC-resistant patients (*n* = 36); normalized according to the GFPT1 levels of GC-sensitive patients; unpaired 2-tailed Student’s *t* test. ***P < 0.001 represent significant differences between 2 groups; ns represents no significant difference.

To determine the changes of metabolic patterns in GC-resistant BCa tissues, we initially conducted metabolomic profiling of tumor tissues from 5 GC-sensitive BCa patients and 5 GC-resistant BCa patients. Interestingly, we found that UDP-GlcNAc, a crucial metabolite in the HBP metabolism, was dramatically up-regulated in GC-resistant patients with the highest fold change (Fig. 1A to C and Fig. S1A). Furthermore, we detected the expression levels of key enzymes in the HBP metabolism (GFPT1, GNA-1, PGM3, and UAP) in post-chemotherapy BCa tissues from 37 GC-sensitive patients and 36 GC-resistant patients. GFPT1 (Fig. 1D), GNA-1 (Fig. S1B), PGM3 (Fig. S1C), and UAP (Fig. S1D) were remarkably up-regulated in GC-resistant BCa tissues, with GFPT1 showing the most significant increase (Fig. S1E). Consistently, the translational levels of GFPT1 and O-GlcNAc were also enhanced in GC-resistant tumor tissues (Fig. 1E and Fig. S1F to J). To investigate the predictive potential of GFPT1, GNA-1, PGM3, and UAP for GC sensitivity, 30 GC-sensitive patients and 30 GC-resistant patients were enrolled as the training set to generate receiver operating characteristic (ROC) curves (Fig. 1F and Fig. S2A to C). We found that the transcriptional levels of GFPT1 in post-chemotherapy tissues accurately reflect the effectiveness of GC therapy. In addition, GFPT1 expression in pre-chemotherapy biopsy tissues was also increased in GC-resistant tumor tissues (Fig. 1G), which can serve as an accurate early screening marker for predicting the sensitivity to GC therapy (Fig. 1H). This finding was corroborated in an independent validation set composed of another 7 GC-sensitive patients and 6 GC-resistant patients (Fig. S2D). These results raised the possibility that high expression of GFPT1 is implicated in the primary resistance. However, subsequent analyses indicated that GC treatment resulted in a substantial rise of GFPT1 in GC-resistant tumor tissues; however, GFPT1 expression was scarcely affected by GC treatment in GC-sensitive tissues (Fig. 1I). These results indicate that GFPT1 up-regulation further decreased sensitivity to GC chemotherapy. In addition, overexpression of GFPT1 in parental (PA) BCa cells rescued the cell viability in response to GC treatment and induced chemoresistance; however, GFPT1 knockout further strengthened the sensitivity to GC treatment (Fig. S2E to G). These results imply that GFPT1 is a crucial switch increasing HBP metabolic flux and thus inducing GC resistance.

### GFPT1 induces chemoresistance by promoting O-GlcNAcylation

**Fig. 2.**
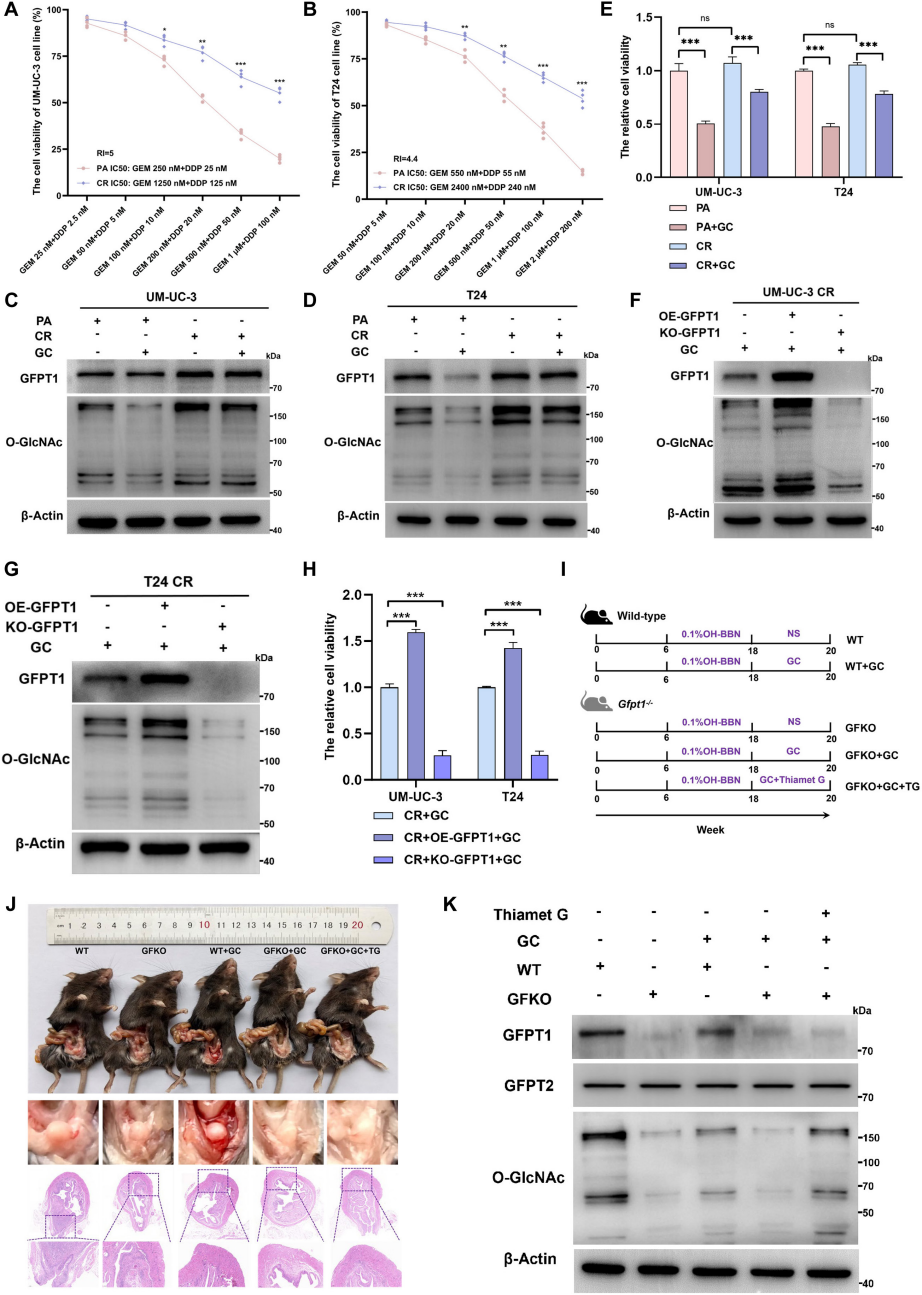
GFPT1 induces O-GlcNAcylation, thus enhancing chemoresistance. (A and B) Cell viability assays showed the cytotoxicity of PA cell lines and CR cell lines after treatments with increasing concentrations of GC for 48 h; the resistance indexes (RI) are 5 and 4.4 in UM-UC-3 CR cell line and T24 CR cell line, respectively; unpaired 2-tailed Student’s *t* test (*n* = 4 per group). (C and D) Levels of GFPT1 and O-GlcNAcylation in PA and CR cell lines treated with GC or not. (E) CCK-8 assays showed cell viabilities of PA and CR cell lines treated with GC or not; normalized according to the cell viability of PA; one-way ANOVA followed by Tukey’s test (*n* = 4 per group). (F and G) Levels of GFPT1 and O-GlcNAcylation in CR cell lines with different GFPT1 levels after GC treatment or not. (H) CCK-8 assays showed cell viabilities of GC-treated CR cell lines with different GFPT1 levels for 48 h; normalized according to the cell viability of CR + GC; one-way ANOVA followed by Tukey’s test (*n* = 4 per group). (I) Schematic graph of the OHBBN-induced orthotopic BCa models and intervention models in GFKO (*Gfpt1^–/–^*) and wild-type (WT) mice (*n* = 6 per group) (gemcitabine 20 mg/kg/2 d; cisplatin 2 mg/kg/2 d). (J) Representative necropsy images (top) and H&E staining (bottom, scale bar: 50 μm) of GFKO and WT mice in the 20th week. (K) Translational levels of GFPT1, GFPT2, and O-GlcNAc in WT and GFKO mice treated with or without GC. ***P < 0.001, **P < 0.01, and *P < 0.05 represent significant differences between 2 groups; ns represents no significant difference.

Then, we constructed 2 types of GC chemoresistance (CR) cell lines (Fig. 2A and B). Compared to PA cell lines, GFPT1 expression and O-GlcNAcylation level were prominently enhanced in CR cells (Fig. 2C and D), along with the increased resistance to GC treatment (Fig. 2E). Knockout of GFPT1 prominently inhibited O-GlcNAcylation and reversed GC resistance in CR cells, while overexpression of GFPT1 decreased the sensitivity to GC treatment (Fig. 2F to H). These findings position GFPT1 as a critical regulator of chemoresistance.

To determine if GFPT1 causes GC resistance by facilitating HBP metabolism-mediated O-GlcNAcylation, we established the orthotopic BCa models in GFPT1^−/−^ (GFKO) mice (Fig. S3A) and wild-type (WT) mice (Fig. 2I). Hematoxylin and eosin (H&E)-stained data showed that the muscle-invasive bladder orthotopic tumor was successfully established in both WT and GFKO mice (Fig. 2J). After 2-week GC treatment, bladder tumors in both WT and GFKO mice were significantly decreased in size, with the more noticeable changes in GFKO mice (Fig. S3B). However, the GC therapeutic effect in GFKO mice was considerably reversed when O-GlcNAcylation was strengthened by Thiamet G (TG) (an OGA inhibitor) (Fig. 2K). In addition, GFPT2, another isoform of GFPT rate-limiting enzyme in the HBP, was not changed in mouse bladder tumor tissues (Fig. 2K) and cell lines (Fig. S3C). Consistently, we did not detect changes in GFPT2 expression between GC-sensitive and GC-resistant BCa tissues (Fig. S3D). These in vitro and in vivo results have provided unequivocal evidence that the up-regulation of GFPT1 promotes chemoresistance by inducing O-GlcNAcylation, and inhibition of GFPT1 and O-GlcNAcylation increases the chemosensitivity.

### NR3C1 O-GlcNAcylation at Thr^299^ facilitates chemoresistance

**Fig. 3.**
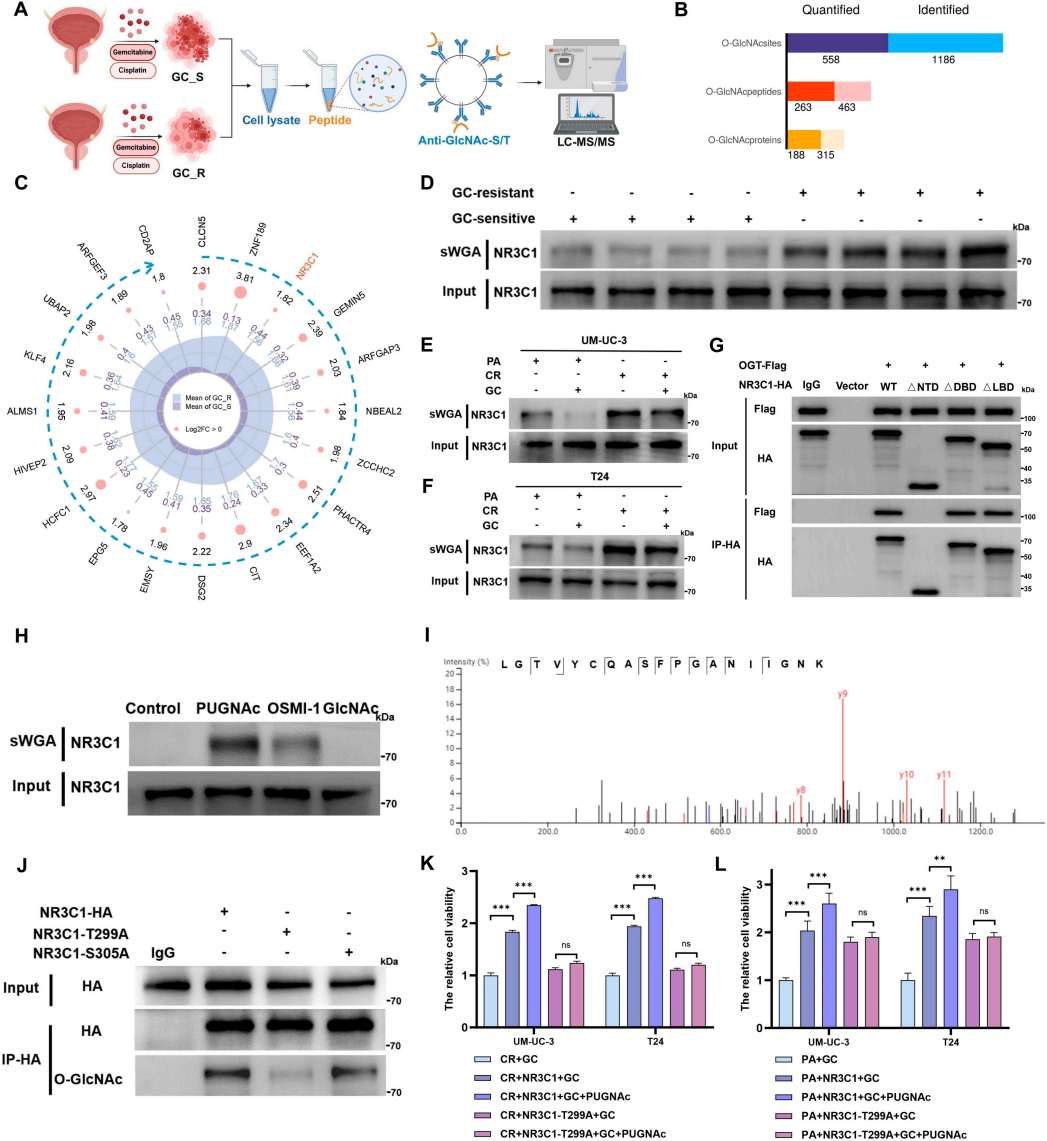
NR3C1 O-GlcNAcylation at Thr^299^ promotes chemoresistance. (A) Schematic diagram of the O-GlcNAcylation omics analysis of GC-sensitive (GC_S) and GC-resistant (GC_R) BCa tissues. (B) Identified and quantified O-GlcNAcylated sites, peptides, and proteins. (C) Radar map showing the top 20 differentially O-GlcNAcylated proteins between GC_S and GC_R BCa tissues; blue dotted arrow directing the O-GlcNAcylated score from high to low. sWGA pull-down assays exhibited the O-GlcNAcylated levels of NR3C1 in BCa tumor samples (D) and 2-type BCa cell lines (E and F). (G) IP assays detecting the interaction between OGT and truncated NR3C1. (H) sWGA pull-down assays were performed in CR cells after treatments with 50 μM PUGNAc or 50 μM OSMI-1 for 24 h; WB assays were detected using an anti-NR3C1 antibody. (I) MS analysis identified potential NR3C1 O-GlcNAcylated site. (J) IP assays using an anti-HA antibody in CR cells transfected with NR3C1-WT-HA or NR3C1-T299A-HA; WB assays were performed using anti-O-GlcNAc and anti-HA antibodies. (K and L) Relative cell viabilities in GC-treated CR cells and PA cells transfected with NR3C1-WT or NR3C1-T299A mutant; normalized according to the levels in CR + GC or PA + GC group; one-way ANOVA followed by Tukey’s test (*n* = 4 per group). ***P < 0.001 and **P < 0.01 represent significant differences between 2 groups; ns represents no significant difference.

Next, we performed global O-GlcNAcylation omics to disclose the differences of O-GlcNAcylated maps between cancer cells isolated from GC-sensitive and GC-resistant tissues (Fig. 3A and B). We identified 1,186 O-GlcNAcylated sites on 315 proteins, of which NR3C1 was highly O-GlcNAcylated in GC-resistant cancer cells (Fig. 3C). Recent evidence suggested that NR3C1 was implicated in chemoresistance, whereas underlying mechanisms are not fully understood [21,23]. Consistently, succinylated wheat germ agglutinin (sWGA) pull-down assays corroborated that NR3C1 O-GlcNAcylation was prominently enhanced in GC-resistant BCa tissues (Fig. 3D) and CR cells (Fig. 3E and F). O-GlcNAc transferase (OGT) is a highly conserved enzyme that catalyzes protein O-GlcNAcylation. The interactions of GR with O-GlcNAc (Fig. S4A) and OGT (Fig. S4B and C) were validated in CR cell lines. Specifically, OGT interacted with the N-terminal domain (NTD) of NR3C1 (Fig. 3G). Besides, NR3C1 O-GlcNAcylation was enhanced by PUGNAc (an OGA inhibitor) but weakened by OSMI-1 (an OGT inhibitor) or GlcNAc in CR cells (Fig. 3H). Next, we confirmed the O-GlcNAcylated sites on NR3C1, which were detected in the O-GlcNAcylation omics analysis, and found that O-GlcNAcylation primarily occurred at the 299th threonine site (Thr^299^) (Fig. 3I and J).

Then, we intend to know if NR3C1 O-GlcNAcylation at Thr^299^ induces chemoresistance. We found that overexpression of NR3C1 decreased chemosensitivity to varying degrees in CR and PA cell lines. Compared to CR cells, overexpression of both NR3C1 WT and Thr^299^ mutant in PA cells demonstrated the more pronounced effects on cell viability, probably because GC chemotherapy caused a notably increased ferroptosis and decreased cell viability in PA cells. Notably, Thr^299^ mutation exerted a comparatively weaker impact on NR3C1-mediated ferroptosis suppression in PA cells with low O-GlcNAcylation levels, thus sustaining significant cell viability in response to GC treatment. In contrast, Thr^299^ site-directed mutagenesis led to a notable reduction of NR3C1 O-GlcNAcylation in CR cells, thereby substantially abrogating NR3C1-mediated cytoprotection. In addition, PUGNAc further augmented the resistance to GC in NR3C1-overexpressed cells, but did not affect the NR3C1 Thr^299^ mutant (Fig. 3J and K). These findings raise an intriguing possibility that NR3C1 O-GlcNAcylation at the Thr^299^ site promotes GC resistance in BCa.

### NR3C1 O-GlcNAcylation at Thr^299^ increases protein stability by attenuating NR3C1 ubiquitination

**Fig. 4.**
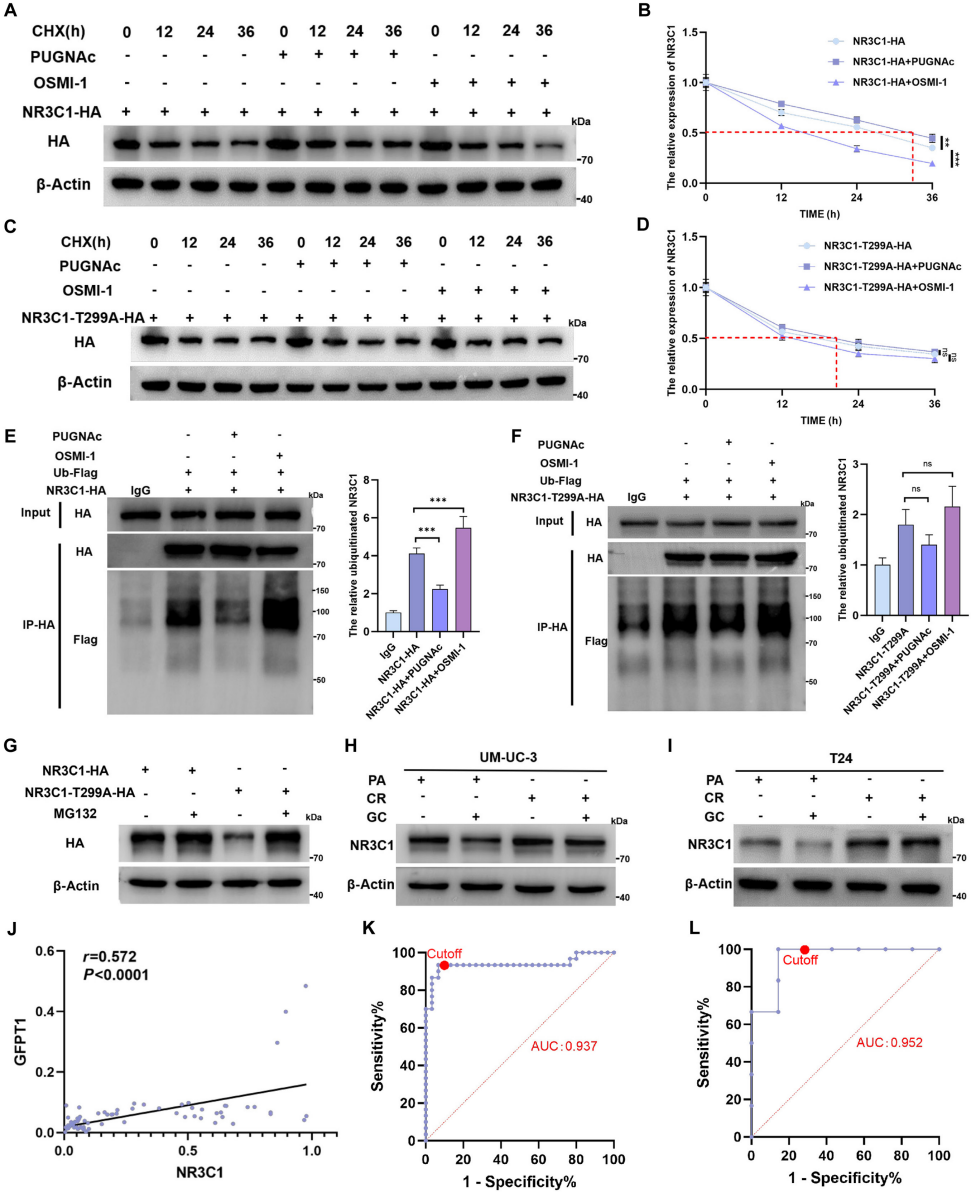
NR3C1 O-GlcNAcylation at Thr^299^ potentiates the protein stability of NR3C1. (A to D) CHX assays and half-life analyses of NR3C1-WT-HA and NR3C1-T299A-HA mutant in CR cells following treatments with 50 μM PUGNAc or 50 μM OSMI-1 for 24 h. The ubiquitination assays of NR3C1-WT-HA (E) and NR3C1-T299A-HA mutant (F) in CR cells transfected with Flag-tagged ubiquitin by using an anti-HA antibody; WB assays were performed using anti-Flag and anti-HA antibodies; Flag-tagged ubiquitin was quantified and normalized according to the levels in IgG group; one-way ANOVA followed by Tukey’s test (*n* = 4 per group). (G) Protein expression of HA-tagged NR3C1 and NR3C1-T299A mutant in CR cells treated with MG132 or not. (H and I) NR3C1 expression in PA and CR cell lines with GC treatment or not. (J) Pearson correlation analysis of NR3C1 and GFPT1 in biopsy BCa tissues (*n* = 73, *r* = 0.572, *P* < 0.0001). (K) Training ROC curve of the early predicting model for GC effects based on NR3C1 levels in biopsy BCa tissues [*n* = 60, area under curve (AUC): 0.937, 95% CI: 0.8653 to 1.000, *P* < 0.001]. (L) Validation ROC curves of the early predicting model based on the NR3C1 levels in BCa biopsy tissues of GC-sensitive patients (*n* = 7) and GC-resistant (*n* = 6) patients (AUC: 0.952, 95% CI: 0.8390 to 1.000, *P* = 0.007). ***P < 0.001 and **P < 0.01 represent significant differences between 2 groups; ns represents no significant difference.

Then, we investigated if NR3C1 O-GlcNAcylation affects protein functions, such as subcellular localization and stability. The immunofluorescence results indicated a colocalization of NR3C1 and OGT in the cytoplasm, but PUGNAc or OSMI-1 did not affect the subcellular localization of NR3C1 (Fig. S4D). Intriguingly, the induction of O-GlcNAcylation significantly increased the translational levels of NR3C1 (Fig. S4E and F), but scarcely showed effects on the transcription levels (Fig. S4G). Then, the cycloheximide (CHX) half-life experiments suggested that, following PUGNAc treatment, NR3C1 was more stable in CR cells with a longer half-life of more than 30 h (Fig. 4A and B). Importantly, the roles of PUGNAc and OSMI-1 on the half-life of NR3C1 were dramatically weakened upon the Thr^299^ mutation (Fig. 4C and D), indicating that NR3C1 O-GlcNAcylation enhances protein stability.

Previous literatures provided the theoretical basis indicating that NR3C1 was primarily degraded through the proteasomal pathway, rather than the lysosomal pathway [24,25]. We subsequently investigated if O-GlcNAcylation increases NR3C1 stability by regulating ubiquitin–proteasome pathway-dependent degradation. The results showed that O-GlcNAcylation inhibited NR3C1 degradation mediated by the ubiquitin–proteasome pathway, evidenced by the alterations of NR3C1 ubiquitination after PUGNAc and OSMI-1 treatments (Fig. 4E). Consistent with the regulating roles on NR3C1 stability, the ubiquitination of Thr^299^ mutant was barely affected by PUGNAc or OSMI-1 (Fig. 4F). Then, we validated the crucial roles of proteasome pathway in NR3C1 degradation by using MG132. We overexpressed NR3C1-WT and NR3C1-T299A mutant in CR cells with MG132 treatment or not. Compared to NR3C1-WT, the protein level of NR3C1-T299A mutant was considerably decreased. Interestingly, inhibition of proteasome-mediated degradation via MG132 rescued the protein level of NR3C1-T299A, nearly restoring to the level of MG132-treated NR3C1-WT group (Fig. 4G). These results further support the role of the proteasome pathway in regulating NR3C1 degradation. Taken together, NR3C1 O-GlcNAcylation at Thr^299^ potentiated protein stability by suppressing NR3C1 ubiquitination and proteasome-dependent degradation.

In addition, we validated that the translational levels of NR3C1 in CR cells were considerably higher than those in PA cells (Fig. 4H and I). Overexpression of NR3C1 increased resistance to GC in both PA and CR cells, while knockout of NR3C1 reversed the GC resistance (Fig. S4H and I). The expression of NR3C1 in 60 BCa biopsy tissues was positively correlated to GFPT1 (Fig. 4J). Intriguingly, consistent with the predicting value of GFPT1, the ROC curves showed that NR3C1 is also a potential predictor for GC sensitivity in BCa (Fig. 4K), which was further validated in an independent validation set (Fig. 4L).

### GFPT1-induced NR3C1 O-GlcNAcylation at Thr^299^ augments transcriptional activity of GPX4 and inhibits GC-induced ferroptosis

**Fig. 5.**
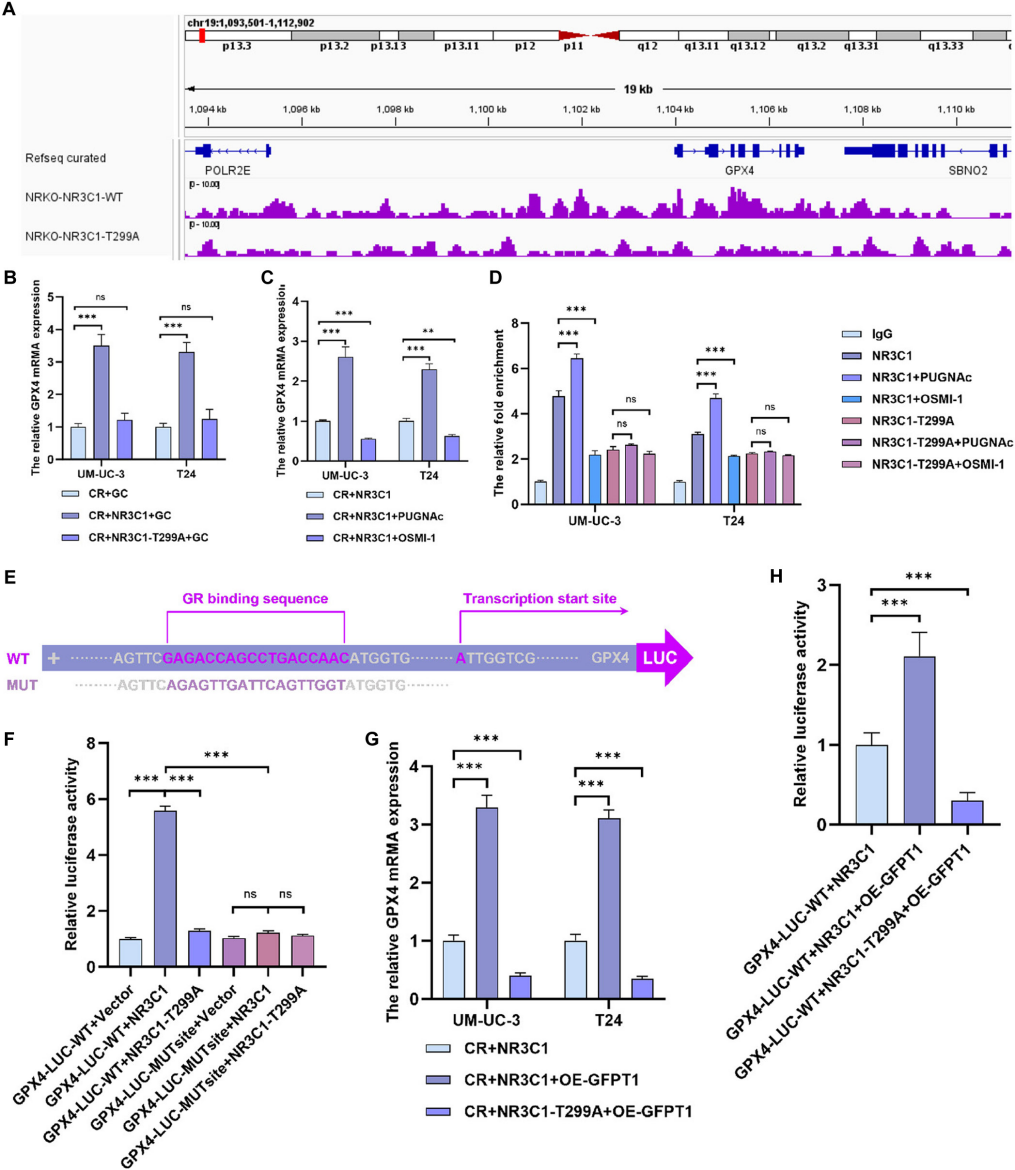
NR3C1-Thr^299^ O-GlcNAcylation promotes GPX4 transcription. (A) ChIP-seq tracks for GPX4 in NR3C1-KO CR cells transfected with NR3C1 or NR3C1-T299A. (B) Transcriptional levels of GPX4 in GC-treated CR cells transfected with NR3C1 or NR3C1-T299A; normalized according to the expression of CR + GC; one-way ANOVA followed by Tukey’s test (*n* = 4 per group). (C) Transcriptional levels of GPX4 in NR3C1-overexpressed CR cells treated with 50 μM PUGNAc (24 h), 50 μM OSMI-1 (24 h), or not; normalized according to the expression of CR + NR3C1; one-way ANOVA followed by Tukey’s test (*n* = 4 per group). (D) ChIP-qPCR assays detecting the binding of NR3C1 and NR3C1-T299A mutant on GPX4 promoter; normalized according to the GPX4 promoter binding level of IgG; one-way ANOVA followed by Tukey’s test (*n* = 4 per group). (E) Schematic of the binding sequences of NR3C1 on GPX4 promoter. (F) Dual-luciferase reporter assays determining the specificity of binding site of NR3C1 on GPX4 promoter; detecting the effect of T299A mutation on luciferase activity; normalized according to the luciferase activity levels of GPX4-LUC-WT + Vector; one-way ANOVA followed by Tukey’s test (*n* = 4 per group). (G) Transcriptional levels of GPX4 in CR cells transfected with NR3C1-WT-HA or NR3C1-T299A-HA and with different GFPT1 expression; normalized according to the expression of CR + NR3C1; one-way ANOVA followed by Tukey’s test (*n* = 4 per group). (H) Dual-luciferase reporter assays determining the effect of GFPT1 on NR3C1-regulated GPX4 transcription; normalized according to the luciferase activity levels of GPX4-LUC-WT + NR3C1; one-way ANOVA followed by Tukey’s test (*n* = 4 per group). ***P < 0.001 represents a significant difference between 2 groups; ns represents no significant difference.

Given the regulatory function of NR3C1 on gene transcription, we then investigate how NR3C1 O-GlcNAcylation induces GC resistance via chromatin immunoprecipitation sequencing (ChIP-seq) analysis. The results indicated a sharp reduction in the binding peaks between NR3C1 and GPX4 promoter in Thr^299^ mutant (Fig. 5A). GPX4, which acts as a crucial regulator in ferroptosis, has been demonstrated to participate in the antineoplastic drug resistance [26]; however, the molecular mechanisms remain unclear. We revealed that overexpression of NR3C1-WT causes a notable increase of GPX4 mRNA level in CR cells, which was reversed upon O-GlcNAcylated site mutation (Fig. 5B). Additionally, up-regulation of NR3C1 O-GlcNAcylation via PUGNAc further increased the transcriptional level of GPX4, while OSMI-1 took an opposite effect (Fig. 5C). We also detected the change of other ferroptosis regulator, such as SLC7A11, and found that NR3C1 did not affect SLC7A11 expression, regardless of PUGNAc and OSMI-1 treatments (Fig. S5A). ChIP-qPCR (quantitative polymerase chain reaction) assays confirmed that PUGNAc increased the interaction between NR3C1-WT and GPX4 promoter, while OSMI-1 reduced this interaction. Compared to NR3C1-WT, the interaction between T299A mutant and GPX4 promoter was decreased and unaffected by PUGNAc and OSMI-1 treatments (Fig. 5D). To further confirm NR3C1-mediated transcriptional activation of GPX4, we established the constructs respectively carrying the WT cDNA sequence of GPX4 promoter and the mutant of the NR3C1 binding site “GAGACCAGCCTGACCAAC” (Fig. 5E and Table S2). The results of dual-luciferase assays showed that NR3C1-WT enhanced the luciferase activity of WT GPX4, rather than the mutant. Importantly, mutation of O-GlcNAcylated site dramatically weakened NR3C1-mediated GPX4 transcription activation (Fig. 5F).

Subsequently, we determine if GFPT1 affects GPX4 expression and the transcriptional activation effect of NR3C1. Overexpression of GFPT1 notably increased the transcriptional level of GPX4 in NR3C1-overexpressing cells, but scarcely affected the NR3C1-T299A mutant (Fig. 5G). Consistently, dual-luciferase assays indicated that GFPT1 strengthened NR3C1-mediated GPX4 transcription activation, while T299A mutation prominently impaired the effect of GFPT1 (Fig. 5H). Collective with these results, we concluded that up-regulation of GFPT1-induced NR3C1 O-GlcNAcylation at Thr^299^ increases its binding to the GPX4 promoter and thus promotes GPX4 transcription.

**Fig. 6.**
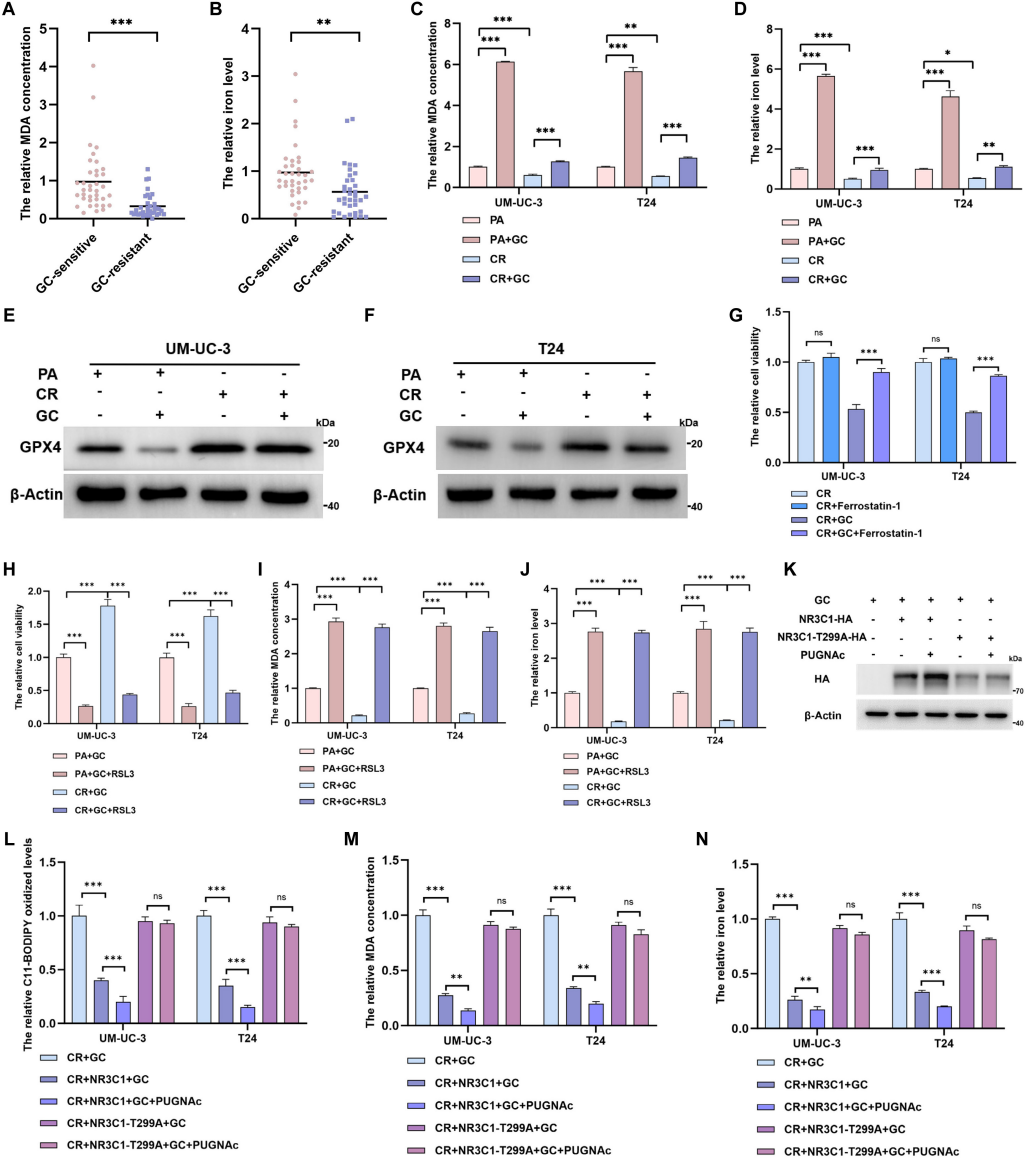
NR3C1 O-GlcNAcylation Thr^299^ facilitates chemoresistance by suppressing GC-induced ferroptosis. The relative MDA concentrations (A) and iron levels (B) in 37 GC-sensitive and 36 GC-resistant BCa tissues; normalized by the levels in GC-sensitive BCa tissues; unpaired 2-tailed Student’s *t* test. The relative MDA concentrations (C) and iron levels (D) in PA and CR cell lines treated with GC (IC_50_ of the PA cell lines) or not; normalized by the levels in PA group; one-way ANOVA followed by Tukey’s test (*n* = 4 per group). (E and F) Protein levels of GPX4 in PA and CR cell lines treated with GC or not. (G) CCK-8 assays detecting the relative cell viabilities of CR cell lines after treatments with GC (IC_50_ of the CR cell lines) and (or) 1 μM ferrostain-1; normalized by the cell viabilities in CR group; one-way ANOVA followed by Tukey’s test (*n* = 4 per group). The relative cell viabilities (H), MDA concentrations (I), and iron levels (J) of GC-treated BCa cell lines administrated with 1 μM RSL3 or not; normalized by the levels in PA + GC group; one-way ANOVA followed by Tukey’s test (*n* = 4 per group). (K) Protein levels of HA-tagged NR3C1-WT and NR3C1-T299A in GC-treated CR cells (IC_50_ of the CR cell lines). Following specified gene intervention (WT or T299A mutation) of NR3C1, CR cells were treated with or without 50 μM PUGNAc for 24 h. Subsequently, the relative lipid peroxidation levels via C11-BODIPY fluorescent probe (L), MDA concentrations (M), and iron levels (N) were conducted; normalized according to the levels in CR + GC group; one-way ANOVA followed by Tukey’s test (*n* = 4 per group). ***P < 0.001, **P < 0.01, and *P < 0.05 represent significant differences between 2 groups; ns represents no significant differences.

Then, we detected that ferroptosis in GC-resistant BCa tissues was significantly decreased, evidenced by the reduction of malondialdehyde (MDA) concentration (Fig. 6A) and iron level (Fig. 6B). Compared to CR cells, the ferroptosis level in PA cells was considerably up-regulated after GC treatment (Fig. 6C and D), consistent with the decrease of GPX4 (Fig. 6E and F). Interestingly, inhibition of ferroptosis by ferrostatin-1 largely reversed GC-induced cell death and decreased the sensitivity to GC treatment in CR cells (Fig. 6G and Fig. S5B and C) and PA cells (Fig. S5D to F). RSL3, a ferroptosis inducer by inactivating GPX4, sharply rose the ferroptosis levels and the sensitivity to GC therapy in CR cells (Fig. 6H to J). These results confirmed that down-regulation of ferroptosis provoked chemoresistance.

Next, we intend to know if NR3C1 O-GlcNAcylation at Thr^299^ regulates ferroptosis and thereby induces chemoresistance. We overexpressed NR3C1-WT and NR3C1-T299A mutant in GC-treated CR cells, and detected a higher protein level of NR3C1-WT than T299A mutant. Additionally, enhancing O-GlcNAcylation via PUGNAc further increased NR3C1 protein expression, but barely affected the T299A mutant (Fig. 6K). Subsequently, we revealed that overexpression of NR3C1-WT significantly inhibited GC-induced ferroptosis in CR cells (Fig. 6L to N) and PA cells (Fig. S6A to C), and PUGNAc further augmented the inhibiting roles of NR3C1 on ferroptosis; however, the NR3C1-T299A mutant had a modest effect on GC-induced ferroptosis, regardless of PUGNAc treatment. Collective with the finding in Fig. 3J and K, we conclude that NR3C1 O-GlcNAcylation at Thr^299^ facilitates chemoresistance by inhibiting ferroptosis.

To determine whether NR3C1 O-GlcNAcylation at Thr^299^ regulates ferroptosis through the O-GlcNAcylation–ubiquitination axis, we generated a ubiquitination-resistant mutant (K419R) [25,27] on both WT and T299A backgrounds (Fig. S6D). Genetic impairment of ubiquitination (via K419R mutagenesis) restored T299A protein levels (Fig. S6E) and abrogated T299A-mediated ferroptosis induction, confirming that NR3C1 O-GlcNAcylation-dependent control of ubiquitination is the primary mechanistic driver of the phenotype (Fig. S6F to H). Therefore, we conclude that the observed divergence in ferroptosis stems predominantly from O-GlcNAcylation deficiency-driven destabilization of NR3C1.

### GFPT1 promotes chemoresistance by regulating ferroptosis

**Fig. 7.**
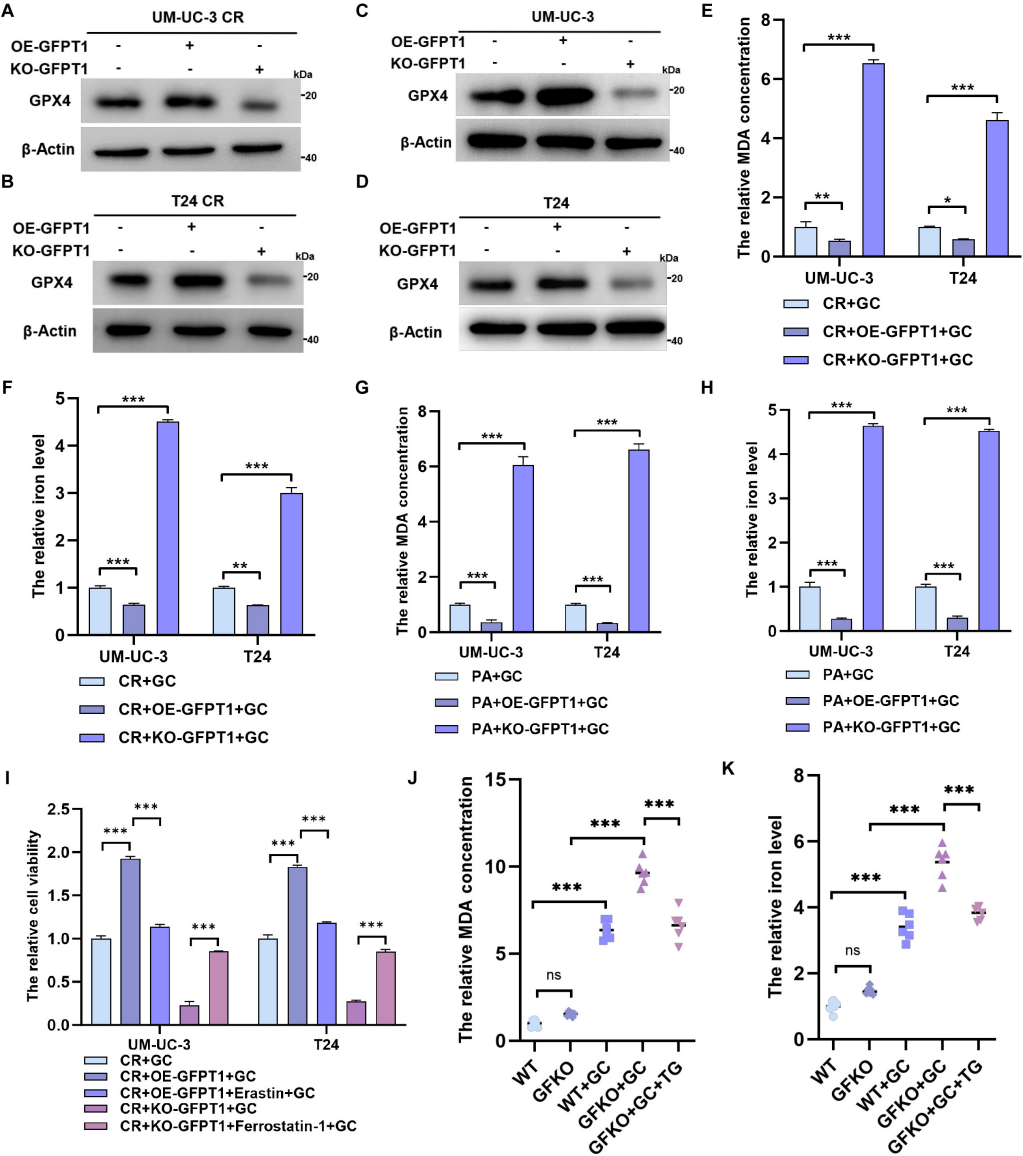
GFPT1 regulates ferroptosis and chemosensitivity. The protein levels of GPX4 in CR (A and B) and PA (C and D) cell lines with different GFPT1 levels. The relative MDA concentrations and iron levels in GC-treated CR (E and F) and PA (G and H) cell lines with different GFPT1 levels; normalized by the levels in CR + GC or PA + GC group; one-way ANOVA followed by Tukey’s test (*n* = 4 per group). (I) CCK-8 assays detecting the relative cell viabilities of GC-treated CR cells with different GFPT1 levels after treatments with 10 μM erastin or 1 μM ferrostain-1; normalized by the levels in CR + GC group; one-way ANOVA followed by Tukey’s test (*n* = 4 per group). The relative MDA concentrations (J) and iron levels (K) in BCa tissues from WT and GFKO mice treated with GC and (or) TG; normalized by the levels in WT group; one-way ANOVA followed by Tukey’s test (*n* = 6 per group). ***P < 0.001, **P < 0.01, and *P < 0.05 represent significant differences between 2 groups; ns represents no significant differences.

We have demonstrated that up-regulation of GFPT1 decreased the sensitivity to GC in CR and PA cells and induced chemoresistance (Fig. 2E and Fig. S2H). Mechanistically, we revealed that overexpression of GFPT1 promoted GPX4 expression (Fig. 7A to D) and inhibited ferroptosis (Fig. 7E to H) in both CR and PA cells, but knockout of GFPT1 took an opposite effect. To determine whether GFPT1 induces GC resistance by suppressing ferroptosis, we separately used the ferroptosis inductor and inhibitor to treat GFPT1-overexpressed cells and GFPT1-knockout cells. We found that overexpression of GFPT1 reduced the sensitivity to GC in CR cells, but the reduction was sharply reversed when ferroptosis was elevated by erastin. Meanwhile, GFPT1 knockout efficiently reversed GC resistance, while inhibition of ferroptosis via ferrostatin-1 restored cell viability in response to GC treatment (Fig. 7I).

Then, we established cell line-derived xenograft mouse models using PA and GFPT1-overexpressed PA cells. Up-regulation of GFPT1 decreased the sensitivity to GC (Fig. S7A to D) and inhibited ferroptosis (Fig. S7E and F), and suppression of ferroptosis also impaired the efficiency of GC. Consistently, the results of *N*-butyl-*N*-4-hydroxybutyl nitrosamine (OHBBN)-induced orthotopic BCa models indicated that knockout of GFPT1 caused a dramatic up-regulation of GC-induced ferroptosis (Fig. 7J and K) and further enhanced the antitumor effect of GC (Fig. S3A and B). However, enhancing O-GlcNAcylation via TG rescues the up-regulation of ferroptosis induced by GFPT1 knockout. These in vitro and in vivo results indicate that GFPT1 promotes chemoresistance by modulating ferroptosis.

### NR3C1 O-GlcNAcylation at Thr^299^ induces chemoresistance in vivo

**Fig. 8.**
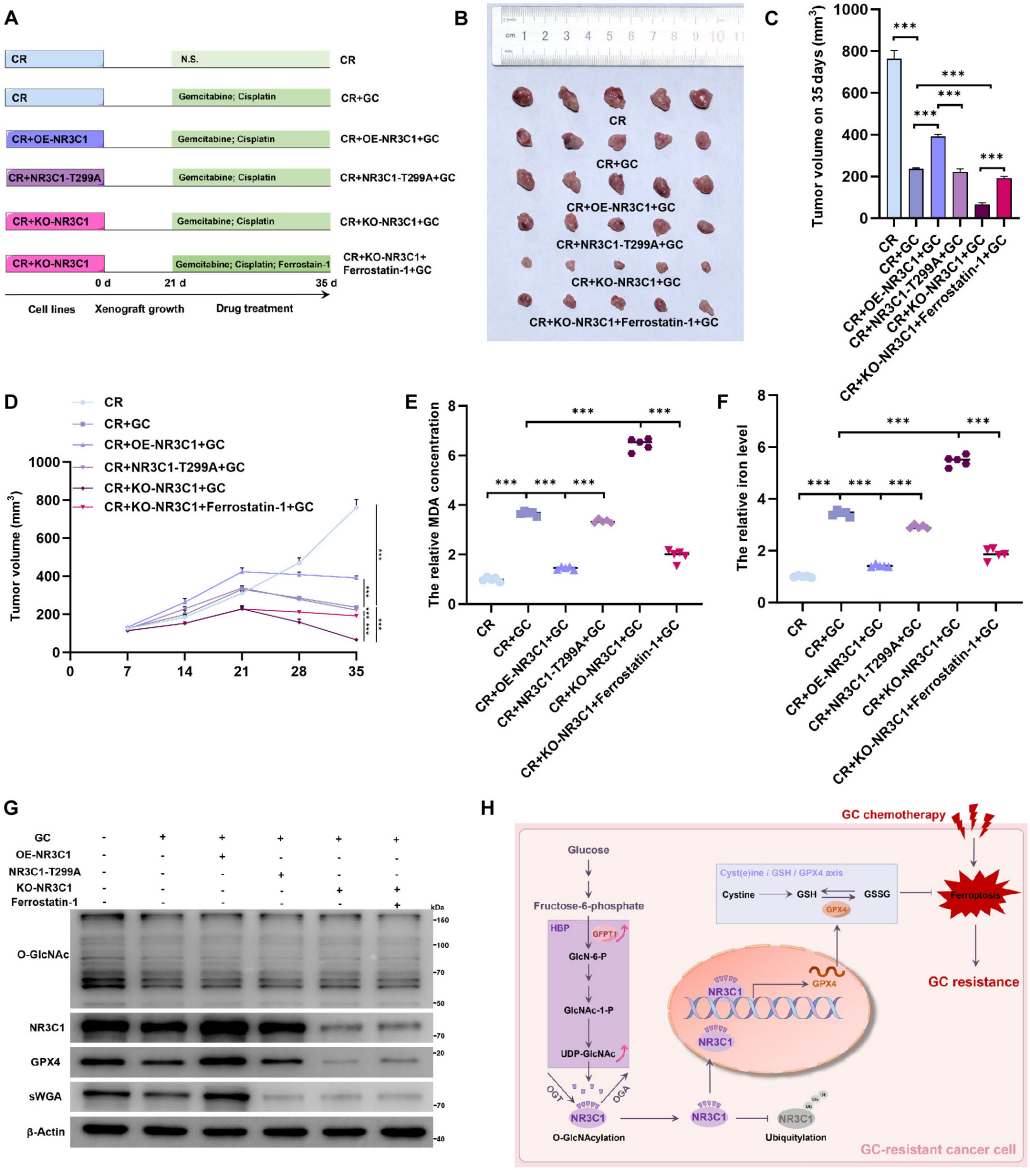
HBP metabolic reprogramming-mediated NR3C1-Thr^299^ O-GlcNAcylation causes chemoresistance by inhibiting ferroptosis. (A) Schematic diagram of the experimental protocols. CR cells with different NR3C1 levels were subcutaneously injected into 5-week-old female BALB/c nude mice to establish CR cell line-derived xenograft mouse models; normal saline, GC (gemcitabine 20 mg/kg/2 d; cisplatin 2 mg/kg/2 d), and (or) ferrostain-1 (5 mg/kg/2 d) were treated from the 8th week (*n* = 5 per group). NS represents mice that were injected with normal saline (*n* = 5 per group). (B) Images of CR cell line-derived xenograft tumors. (C) Tumors were weighted on the 35th day after xenograft; one-way ANOVA followed by Tukey’s test (*n* = 5 per group). (D) Tumor volumes were measured on the 7th, 14th, 21st, 28th, and 35th day after xenograft; one-way ANOVA followed by Tukey’s test (*n* = 5 per group). The relative MDA concentration (E) and iron level (F) in xenograft tissues; normalized by the levels of CR; one-way ANOVA followed by Tukey’s test (*n* = 5 per group). (G) WB and sWGA pull-down assays detected the levels of NR3C1, GPX4, O-GlcNAc, and NR3C1 O-GlcNAcylation in xenograft tissues. (H) Mechanism diagram of NR3C1 O-GlcNAcylation causes GC resistance by inhibiting GC-induced ferroptosis in BCa. ***P < 0.001 represents a significant difference between 2 groups.

We then established CR cell line-derived xenograft mouse models to further validate the above findings (Fig. 8A). We found that overexpression of NR3C1 significantly enhanced GC resistance in BCa, which was reversed upon the mutation of Thr^299^. Besides, knockout of NR3C1 improved the chemosensitivity but intraperitoneal injection of ferrostatin-1 unequivocally reversed the efficiency of GC (Fig. 8B to D). Consistently, ferroptosis was considerably attenuated when NR3C1 was overexpressed, while Thr^299^ mutation and knockout of NR3C1 led to an increase of ferroptosis (Fig. 8E and F). Mechanistically, the suppression of NR3C1 O-GlcNAcylation decreases the NR3C1 and GPX4 levels, thus promoting ferroptosis and increasing chemosensitivity (Fig. 8G).

Taken together, these results suggest that GFPT1-triggered HBP metabolic reprogramming facilitates NR3C1 O-GlcNAcylation at Thr^299^ and potentiates its transcriptional activation to GPX4, thereby suppressing ferroptosis and causing chemoresistance (Fig. 8H). Targeted inhibition of GFPT1 and NR3C1 O-GlcNAcylation confers the therapeutic potential for mitigating chemoresistance.

## Discussion

Neoadjuvant chemotherapy is one of the primary strategies for MIBC, but the objective remission rate and 5-year survival rate remain unsatisfactory owing to chemoresistance [2,28]. Mounting evidence disclose the close associations between ferroptosis and chemoresistance [29]. Activation of ferroptosis-related signaling pathways represents a viable strategy to mitigate chemoresistance. For instance, the induction of ferroptosis by inhibiting the NFE2 like BZIP transcription factor 2 (Nrf2) signaling pathway sensitizes resistant gastric cancer cells to cisplatin [30]. Additionally, Qi et al.’s study [10] revealed that CAFs inhibit ferroptosis in pancreatic cancer cells by secreting extracellular vesicles delivering ACSL4-targeted miRNAs, inducing gemcitabine resistance. Ferroptosis is mechanistically diverse in different cellular stress status. It is interesting to investigate the roles and mechanisms of ferroptosis in GC resistance. Herein, we reveal a novel metabolic and epigenetic mechanism underlying ferroptosis in response to GC chemotherapy. A prominent increase of GFPT1 triggers HBP metabolic reprogramming and promotes NR3C1 O-GlcNAcylation at Thr^299^. NR3C1 O-GlcNAcylation improves GPX4 transcriptional activity and inhibits ferroptosis by increasing NR3C1 stability and its binding to GPX4 promoter. Collectively, this study deciphers that the GFPT1/NR3C1/GPX4 axis is a new negative regulatory pathway regulating ferroptosis and inducing chemoresistance.

Complex and plastic metabolic patterns are hallmarks of cancer cells. Metabolic reprogramming satisfies the considerable energy and biosynthesis requirements of tumor cells, facilitating their malignant transformation, cell proliferation, and resistance acquisition [31,32]. In addition, diverse metabolites shape oncogenic signaling pathways and provides crucial substrate for epigenetic modification [33]. Glycolytic reprogramming facilitated lactate accumulation and meiotic recombination 11 homolog 1 (MRE11) lactylation, thereby provoking resistance to olaparib and cisplatin in colon cancer [34]. Low level of glutamine metabolism considerably induced histone H3K27 hypermethylation and decreased sensitivity to PLX4032, a B-Raf proto-oncogene BRAF inhibitor [35]. HBP metabolism remodeling fuels cathepsin B (CTSB) O-GlcNAcylation and promotes pro-tumor polarization of tumor-associated macrophages by inducing CTSB secretion, thus mediating chemoresistance [36]. In the current study, we demonstrated that HBP metabolism is notably up-regulated in GC-resistant cancer cells, leading to increased O-GlcNAcylation levels. Using combinational analysis of metabolomic and O-GlcNAcylation omics, we first unraveled that NR3C1 is highly O-GlcNAcylated at Thr^299^ in response to GC treatment. These findings uncover a previously uncharacterized link of metabolic reprogramming and epigenetic modifications in regulating chemoresistance. However, whether and how other differential metabolites and related epigenetic modifications are implicated in chemoresistance remains an interesting area for further research.

GFPT1 and GFPT2 are the 2 isoforms of GFPT rate-limiting enzymes in the HBP metabolism, with different tissue distribution specificity [37,38]. However, the reason for the distribution specificity remains unknown. In lung adenocarcinoma, GFPT2, but not GFPT1, correlates with poor clinical outcomes [39,40]; however, GFPT1 is required for glycosylation and high expression of GFPT1 predicts poor prognosis in pancreatic ductal adenocarcinoma [41]. In this study, we revealed that GFPT1 plays a crucial role in chemoresistance of BCa by promoting NR3C1 O-GlcNAcylation. Meanwhile, the distribution specificity and the interplay between GFPT1 and GFPT2 are important areas worthy of further research, particularly in the context of tumorigenesis and development.

HBP metabolism-induced O-GlcNAcylation serves as an important nutrient sensor and is highly dynamic in response to diverse cellular stresses [42]. Dysregulation of O-GlcNAcylation takes different regulatory effects on ferroptosis [43]. Yes1-associated transcriptional regulator (YAP) O-GlcNAcylation facilitates transferrin receptor transcription and induces ferroptosis by increasing iron concentration in liver cancer cells [44]. Zinc finger E-box binding homeobox 1 (ZEB1) O-GlcNAcylation at serine 555 promoted protein stabilization and nuclear translocation, which induced lipid peroxidation-dependent ferroptosis [45]. Our previous study demonstrated that HBP metabolism-induced BACH2 O-GlcNAcylation facilitated ferroptosis by suppressing SLC7A11 transcription in renal tubular epithelial cells [14]. Different from the above studies, we unravel a new metabolic link between GFPT1/HBP metabolism and ferroptosis via NR3C1 O-GlcNAcylation at Thr^299^ in this study. The T-to-A mutation mimics the de-O-GlcNAcylated state of NR3C1, leading to a dramatic up-regulation of ferroptosis and thereby alleviating GC resistance in BCa. The inhibiting role of O-GlcNAcylation in ferroptosis was also demonstrated in ischemia–reperfusion–pulmonary epithelial cells [46].

In the current study, we systematically demonstrate that the GFPT1/NR3C1/GPX4 axis plays a critical role in chemoresistance by inhibiting ferroptosis. We discover the novel chemoresistant switch GFPT1, which triggers HBP metabolic reprogramming, facilitates NR3C1 O-GlcNAcylation, activates GPX4 transcription, and inhibits ferroptosis. Targeted inhibition of GFPT1 and NR3C1 O-GlcNAcylation resensitizes the resistant cancer cells to chemotherapy in BCa orthotopic and xenograft mouse models. Additionally, we establish a predicting model based on the GFPT1 and NR3C1 levels in pre-chemotherapy biopsy tissues for chemoresistance. While our findings propose a new metabolic and epigenetic mechanism underlying ferroptosis and GC chemoresistance, certain limitations should be considered. (a) Expanding the use of primary resistance models will enable a more comprehensive investigation into the roles of GFPT1-faciliated NR3C1 O-GlcNAcylation in GC chemoresistance. (b) Cisplatin-based chemotherapy exerts its antitumor effects by orchestrating diverse cell death modalities and distinct molecular pathways, and dysregulation of apoptosis, ferroptosis, and necroptosis could induce cisplatin chemoresistance. In addition, whether other ferroptosis-associated genes are potentially involved in NR3C1 O-GlcNAcylation-mediated suppression of ferroptosis also warrants further investigation.

## Materials and Methods

### Chemicals

PUGNAc (A7229), TG (SML0244), ferrostatin-1 (SML0583), MG132 (M7449), CHX (5087390001), and OSMI-1 (SML1621) were obtained from Sigma (USA). Recombinant PNGaseF (Glycerol-free) (P0709S) was obtained from New England Biolabs (USA). Erastin (HY-15763), gemcitabine (HY-17026), and cisplatin (HY-17394) were obtained from MCE (USA).

### Cell lines and treatments

HEK-293T, UM-UC-3, and T24 cell lines were obtained from the Cell Bank of the Chinese Academy of Sciences (Shanghai, China) and cultured in Dulbecco’s modified Eagle’s medium (Gibco, USA) containing 10% fetal bovine serum (Procell, China) and 1% penicillin and streptomycin (Beyotime, China). To establish GC-resistant BCa cell lines (CR), WT T24 and UM-UC-3 cell lines (PA) were exposed to gradually increasing doses of GC continuously for 6 months, resulting in CR cells capable of surviving at half of the GC IC_50_ (median inhibitory concentration) concentration (UM-UC-3 IC_50_: gemcitabine 200 nM; cisplatin 20 nM; T24 IC_50_: gemcitabine 500 nM; cisplatin 50 nM). In subsequent experiments, we constructed GC treatment cell models using the GC IC_50_ concentrations for PA and CR cells, respectively.

### Clinical samples and clinical response evaluation

A total of 37 GC-sensitive and 36 GC-resistant BCa tissue samples (Table) were obtained with the approval of the Ethics Review Committee of the First Affiliated Hospital of Chongqing Medical University (2020-66). Patients were made clinical response evaluation through at least twice computed tomography scans before and after neoadjuvant chemotherapy. The chemotherapeutic response was assessed based on the reduction of tumor size after GC treatment [47–49]. Following the RECIST 1.1 criteria, patients achieving a partial response (the diameters of target lesions decreased by more than 30%) or better were classified as GC-sensitive (complete response and partial response), whereas those who do not meet these criteria are defined as GC-resistant (stable disease and progressive disease). All patients provided informed consent. All patients had received GC treatment and underwent postoperative diagnosis by the Pathology Department of the First Affiliated Hospital of Chongqing Medical University. Samples were collected and immediately frozen in liquid nitrogen for subsequent experiments.

**Table.** Clinical characteristics of BCa patients

Parameter	GC-sensitive	GC-resistant	*P* value
Number	37	36	
Gender			0.416
Male	24	20	
Female	13	16	
Age (years)			0.393
<65	20	23	
≥65	17	13	
Nidus			0.678
Single	34	31	
Multiple	3	5	
Grade			0.699
High	33	30	
		27	
Low	4	6	
		37	
T stage			0.515
T1	0	0	
		19	
T2	31	28	
		34	
T3	6	8	
		11	
T4	0	0	
Lymph node status			0.981
Negative	36	34	
Positive	1	2	
Distant metastasis site			1
Negative	37	36	
		62	
Positive	0	0	


### Animal experiments

All animal experiments were performed based on protocols approved by the Animal Care and Use Committee and Institutional Review Board (2021-277). This study adhered to all ethical regulations associated with animal studies. C57BL/6-*Gfpt1^+/−^* mice obtained from Association for Assessment and Accreditation of Laboratory Animal Care (AAALAC)-accredited SHANGHAI MODEL ORGANISMS (Shanghai, China) were crossed to construct *Gfpt1^−/−^* (GFKO) mice. Female C57BL/6 and GFKO mice aged 6 weeks were used to establish orthotopic BCa mouse model by drinking 0.1% OHBBN (TCI, Japan) water for 12 weeks. Following that, orthotopic BCa mice were administered GC (gemcitabine 20 mg/kg, cisplatin 2 mg/kg) and TG (20 mg/kg) every 2 d. Female athymic BALB/c nude mice were purchased from Beijing HFK Bio-Technology. A total of 10^6^ UM-UC-3 CR cells resuspended in 100 μl of normal saline were subcutaneously injected into the flank of the right thigh of nude mice. Tumor progression was monitored by weekly measurements until the endpoint. When tumor volumes reached 200 to 400 mm^3^, treatments with normal saline, GC (gemcitabine 20 mg/kg, cisplatin 2 mg/kg), and ferrostatin-1 (5 mg/kg) were administered every 2 d. Tumor volumes were measured weekly until the endpoint.

### Construction of overexpression and knockout cell lines

The cDNA of NR3C1 and NR3C1-T299A was cloned into the lentiviral expression vector pLVX-puro. KO-NR3C1 (GCCAAATCAGCCTTTCCTCG) cell lines were established using the CRISPR/Cas9 system. Subsequently, transfection was performed by polybrene, followed by puromycin selection to obtain stable cell lines. The OE-GFPT1 and KO-GFPT1 cells were described in our previous publication [50].

### Metabolite detection and analysis

Isolated GC-resistant and GC-sensitive BCa tissues were initially washed twice with precooled sodium thiosulfate and then suspended in 500 μl of ice-cold methanol–acetonitrile mixture to extract metabolites and remove proteins. After centrifugation at 14,000*g* at 4 °C for 20 min, 300 μl of supernatant was transferred to a new 1.5-ml Eppendorf (EP) tube and incubated at −20 °C for 30 min. Followed by another centrifugation at 14,000*g* at 4 °C for 10 min, 200 μl of supernatant was subjected to liquid chromatography–mass spectrometry (LC-MS) analysis using a protein precipitation plate. The LC–electrospray ionization (ESI)–MS/MS system (ultra performance liquid chromatography, ExionLC AD; MS, QTRAP 6500+system, https://sciex.com/) was used for metabolite detection and untargeted metabolomic profiling as previously described [14,51].

### MDA and iron assays

The MDA and iron levels in GC-sensitive and GC-resistant BCa tissues, PA cells, and CR cells were measured by using the Lipid Peroxidation MDA Assay Kit (S0131S, Beyotime) and Ferrous Iron Colorimetric Assay Kit (E-BC-K773-M, Elabscience), following the manufacturer’s instructions.

### Lipid peroxidation assay

In the lipid peroxidation assay, cells were plated at a density of 1 × 10^5^ cells per well in 12-well plates and cultured overnight. After treatments, the cells were harvested by trypsinization, washed, and resuspended in phosphate-buffered saline (PBS). Cells were incubated with C11-BODIPY 581/591 (5 μM final concentration) at 37 °C for 30 min, washed twice with ice-cold PBS, and resuspended in fresh PBS for flow cytometry analysis. Data analysis was performed using FlowJo software.

### Cell viability

Cell viability was assessed using Cell Counting Kit-8 (CCK-8, Dojindo, Japan) as previously described [52]. PA and CR cells were seeded in 96-well plates at a density of 5 × 10^3^ cells per well. Subsequently, 10 μM erastin, 1 μM ferrostatin-1, 50 μM PUGNAc, or 50 μM OSMI-1 were administered in the medium for 24 h, followed by treatment with GC for an additional 24 h or no treatment. Then, the cells were incubated with CCK-8 reagent at a 10% concentration in a complete medium for 2 h, and absorbance was measured at 450 nm using a microplate reader (TECAN, USA).

### Immunofluorescence

The pretreated cells were fixed with 4% formaldehyde (Biosharp, China) for 20 min and permeabilized with Triton X-100 (Beyotime, China) for 10 min, followed by PBS washing. Immunol Staining Blocking Buffer (Beyotime, China) was used to block nonspecific antigen for 15 min. Then, cells were incubated overnight at 4 °C with anti-OGT (1:1,000, ab96718, Abcam) and anti-HA (hemagglutinin) (1:1,000, M20003, Abmart) antibodies, followed by incubation with anti-rabbit Alexa Fluor 488 or anti-mouse Alexa Fluor 594 secondary antibodies (1:500) for 1 h. The images were acquired using a laser confocal microscope (Leica Microsystems AG).

### Immunoprecipitation

For immunoprecipitation (IP) assays, NR3C1-HA or NR3C1-T299A-HA and OGT-Flag or Ub-Flag were cotransfected into CR cells using Lipofectamine 3000 (Invitrogen, USA). The pretreated cells were lysed in NP-40 buffer (Beyotime, China) containing protease and phosphatase inhibitors (Bimake, China). Cell lysate was incubated with 2.5 μg of species-matched immunoglobulin G (IgG) or equal amounts of anti-HA or anti-Flag antibodies overnight at 4 °C, followed by 3 washes with IP buffer. Subsequently, cell lysate was incubated with 30 μl of protein A/G magnetic beads (MCE, China) at 4 °C for 6 h. Next, magnetic beads were washed thrice using NP-40 lysis buffer and then boiled in 1× sodium dodecyl sulfate (SDS) loading buffer (Beyotime, China) for Western blot (WB) analyses.

### Chromatin immunoprecipitation

ChIP assays were performed following the manufacturer’s instructions (EZ-CHIP, Merck, Germany). In brief, CR cells with or without 50 μM PUGNAc and 50 μM OSMI-1 treatment, expressing NR3C1-HA or NR3C1-T299A-HA, were cross-linked with 1% formaldehyde for 10 min to stabilize DNA–protein complexes. Subsequently, cells were lysed using SDS lysis buffer containing 1 mM phenylmethylsulfonyl fluoride and a 1% cocktail of protease inhibitors (Sigma, USA). The chromatin was then incubated overnight at 4 °C with anti-HA antibody (3724, CST, USA) or rabbit IgG to precipitate DNA–protein complexes using protein G-agarose beads. The immunoprecipitated DNA was subjected to sequencing analysis and qPCR. The primer sequence of GPX4 promoter was 5′-AATCCAAACCCCTGCCTGTA-3′ (forward) and 5′-CGCGGTATGTGCTCAGAAAA-3′ (reverse).

### Western blot

Total proteins of cells and tumor tissues were obtained using radioimmunoprecipitation assay buffer containing 1% protease inhibitor (Beyotime, China). Equal amounts of proteins were separated by SDS-PAGE (polyacrylamide gel electrophoresis) and transferred onto polyvinylidene difluoride membranes (Millipore, USA), followed by antibody reactions with the following primary antibodies: anti-GFPT1 (1:2,000, ab125069, Abcam), anti-O-GlcNAc (1:1,000, ab2739, Abcam, USA), anti-GPX4 (1:1,000, 381958, Zenbio, China), anti-NR3C1 (1:1,000, 24050-1-AP, Proteintech, China), anti-HA (1:1,000, 3724, CST, USA), anti-Flag (1:1,000, 14793, CST, USA), anti-β-actin (1:10,000, 66009-1-Ig, Proteintech, China), overnight at 4 °C. Subsequently, the membranes were incubated with secondary antibodies conjugated with horseradish peroxidase against mouse or rabbit for 1 h at room temperature. All immunoblot images were captured using the VILBER FUSION FX5 system and analyzed using ImageJ (NIH, USA).

### Real-time qPCR

Total RNA was extracted from pretreated cells by using Trizol (ABclonal, China), and cDNA was prepared using the high-capacity ABScript III RT Master Mix for qPCR with gDNA (ABclonal, China). Subsequently, real-time PCRs were conducted on an ABI-7500 system using SYBR Green reaction mix (ABclonal, China). The expression level was calculated using the equation 2^−ΔΔCT^ and normalized according to β-actin. The primer sequences are listed in Table S1.

### O-GlcNAcylation LC-MS/MS omics

Total proteins of GC-sensitive and GC-resistant BCa tissues were extracted using 8 M urea buffer containing100 mM tris–HCl. Peptides were generated by digestion with 2% trypsin at 37 °C overnight. The resulting peptides were reconstituted in 1.4 ml of prechilled PTMScan Immunoaffinity Purification buffer (CST, USA) and subjected to the enrichment of O-GlcNAc-modified peptides by PTMScan O-GlcNAc [GlcNAc-S/T] Motif Kit (CST, USA). TimsTOF Pro (Bruker, Germany) was used to analyze LC-MS/MS. The original MS data were identified and quantified by Peaks software (BSI, Canada).

### Dual-luciferase reporter assay

The GPX4 promoter was cloned into the pGL3-basic reporter gene vector (Genecreate, China). The NR3C1 binding site on GPX4 promoter was mutated (Table S2). The constructed GPX4-WT or GPX4-MUT plasmids and NR3C1 plasmids were transfected into 293T cells, and the Dual-Luciferase Reporter Assay System (Promega, USA) was used to measure the luciferase activity, which was normalized to Renilla luciferase activity.

### sWGA pull-down assay

BCa cells and tissues were lysed in NP-40 buffer (Beyotime, China). The lysate was denatured in glycoprotein denaturing buffer (NEB, USA), followed by incubation with PNGase (NEB, USA). Then, the lysate was incubated with sWGA biotin-conjugated beads (Vector Laboratories, USA) at 4 °C overnight. The precipitated complex was eluted and analyzed using WB assays.

### Statistical analysis

The ROC package of R language was used to analyze the accuracy of the early prediction model for GC sensitivity based on the expression levels of GFPT1, GNA-1, PGM3, UAP, and NR3C1. SPSS 21.0 and GraphPad Prism 8.0 were used for data analysis. Data are presented as mean ± standard deviation (SD). Comparisons of 2 groups were performed using 2-tailed Student’s *t* test. Comparisons between multiple groups were determined through one-way analysis of variance (ANOVA) followed by Tukey’s multiple comparisons test.

## Ethical Approval

Informed written consents were obtained from all patients, and their personal information was kept confidential. This study was approved by the Medical Ethics Committee of the First Affiliated Hospital of Chongqing Medical University (IRB:2020-66).

## Data Availability

The authors can provide all datasets analyzed during the study on reasonable requirements.
