# Supplementary material for: Combinational Analysis of Metabolomic and O-GlcNAcylation Omics Reveals the HBP Metabolic Regulation of Chemoresistance via GFPT1/NR3C1 O-GlcNAcylation/GPX4 Axis
Source: Research (Wash D C). 2025 Jul 30;8:0809. doi: 10.34133/research.0809 (PMC12308066; doi:10.34133/research.0809)
Supplement: Supplementary 1 — Figs. S1 to S7 Tables S1 and S2 [file research.0809.f1.pdf]

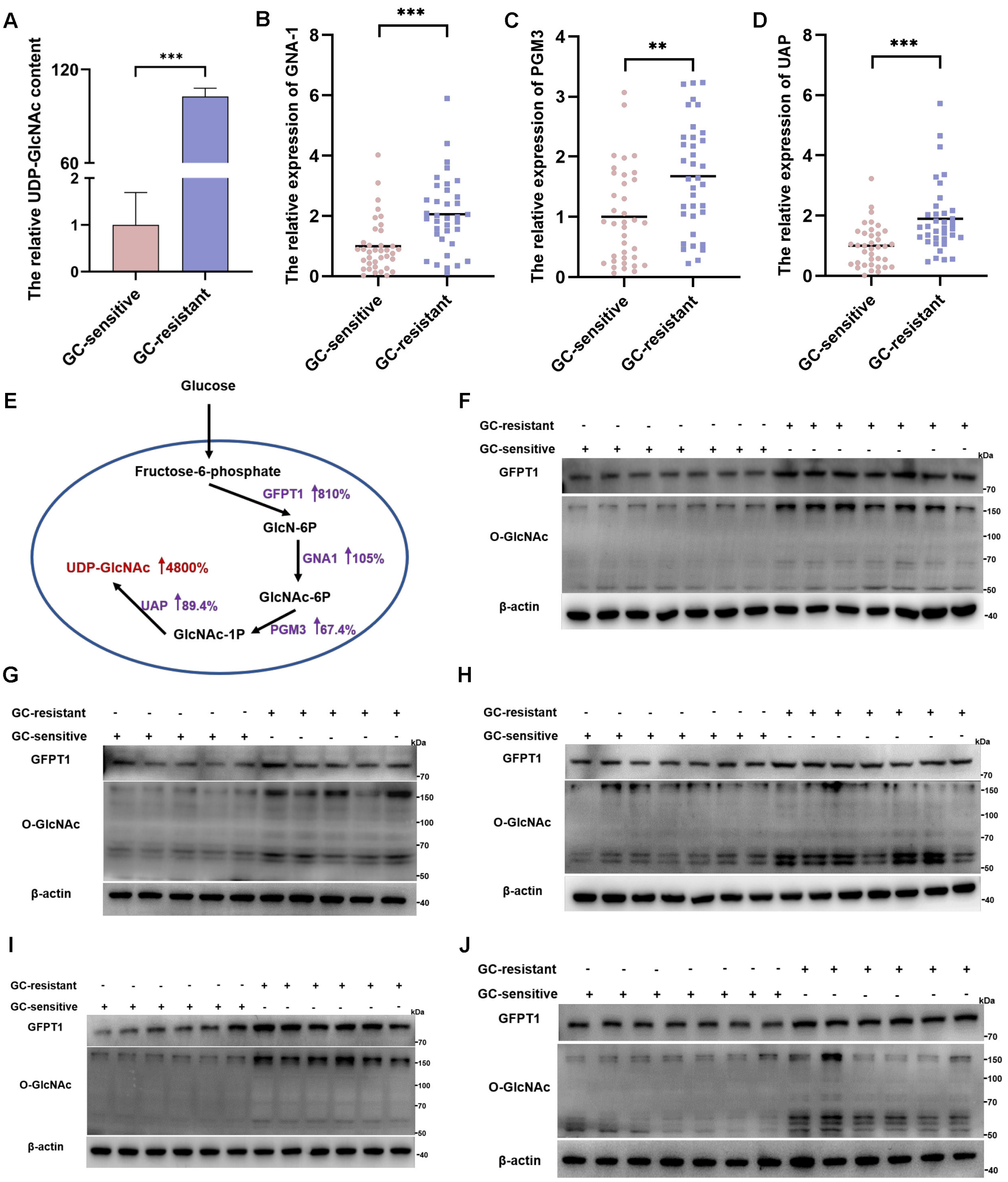

Supplementary Figure 1. GFPT1 induces HBP metabolic reprogramming and facilitates global O-GlcNAcylation and in GC-resistant patients. A. The relative quantitative analysis of UDP-GlcNAc in GC-sensitive and GC-resistant patients; normalized by the GC-sensitive group; unpaired two-tailed Student's t-test (n=5/group). The relative GNA-1 (**B**), PGM3 (**C**) and UAP (**D**) transcriptional levels in BCa tissues of GC-sensitive patients (n = 37) and GC-resistant patients (n =36); the GNA-1, PGM3 and UAP levels were normalized according to the expression levels of GC-sensitive patients; unpaired two-tailed Student's t-test. **E.** Schematic described the details of the HBP metabolic reprogramming in GC-resistant patients. **F-J.** The protein levels of GFPT1 and O-GlcNAc in post-chemotherapy BCa tissues of GC-sensitive and GC-resistant patients. ***$p < 0.001$ and **$p < 0.01$ represent significant differences between two groups.

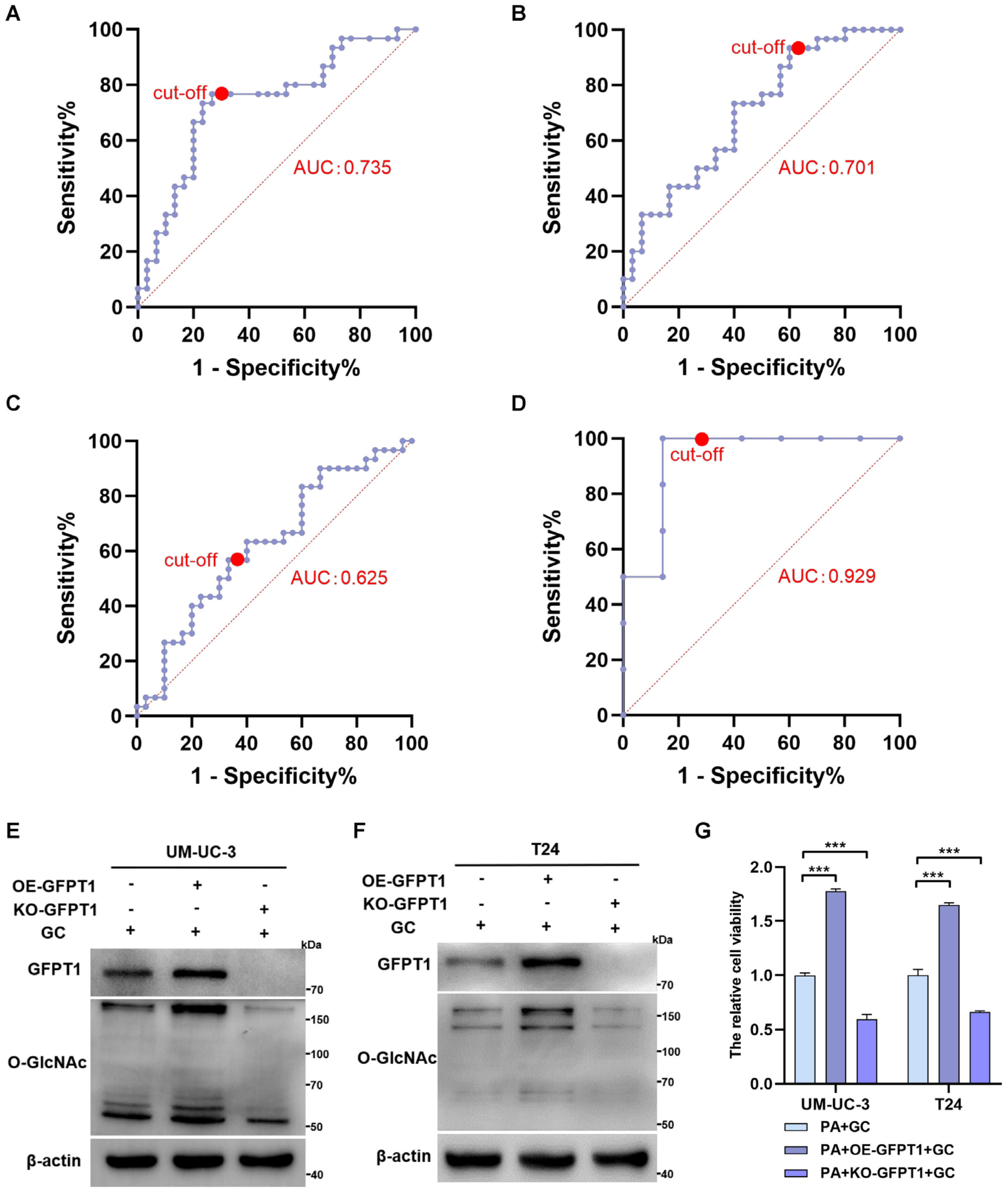

Supplementary Figure 2. High expression GFPT1 is implicated in GC resistance. The ROC curves of the early predicting models for GC effects based on the expression levels of GNA-1 (AUC: 0.735, 95%CI: 0.6060 to 0.8651, $p = 0.002$) (**A**), PGM3 (AUC: 0.701, 95%CI: 0.5700 to 0.8322, $p = 0.008$) (**B**) and UAP (AUC: 0.625, 95%CI: 0.4832 to 0.7679, $p = 0.095$) (**C**) in BCa tissues of GC-sensitive patients (n = 30) and GC-resistant (n = 30) patients. **D.** The validation ROC curves of the early predicting model based on the expression level of GFPT1 in BCa biopsy tissues of GC-sensitive patients (n = 7) and GC-resistant (n = 6) patients (AUC: 0.929, 95%CI: 0.7774 to 1.000, $p = 0.010$). **E-F.** The protein levels of GFPT1 and O-GlcNAc in OE-GFPT1, KO-GFPT1 and PA BCa cell lines treated with GC for 48 h. **G.** CCK-8 assays showed cell viabilities in OE-GFPT1, KO-GFPT1 and PA BCa cell lines treated with GC for 48 h; normalized according to the cell viabilities of PA; one-way ANOVA followed by Tukey's test (n=4/group). ***$p < 0.001$ represents a significant difference between two groups.

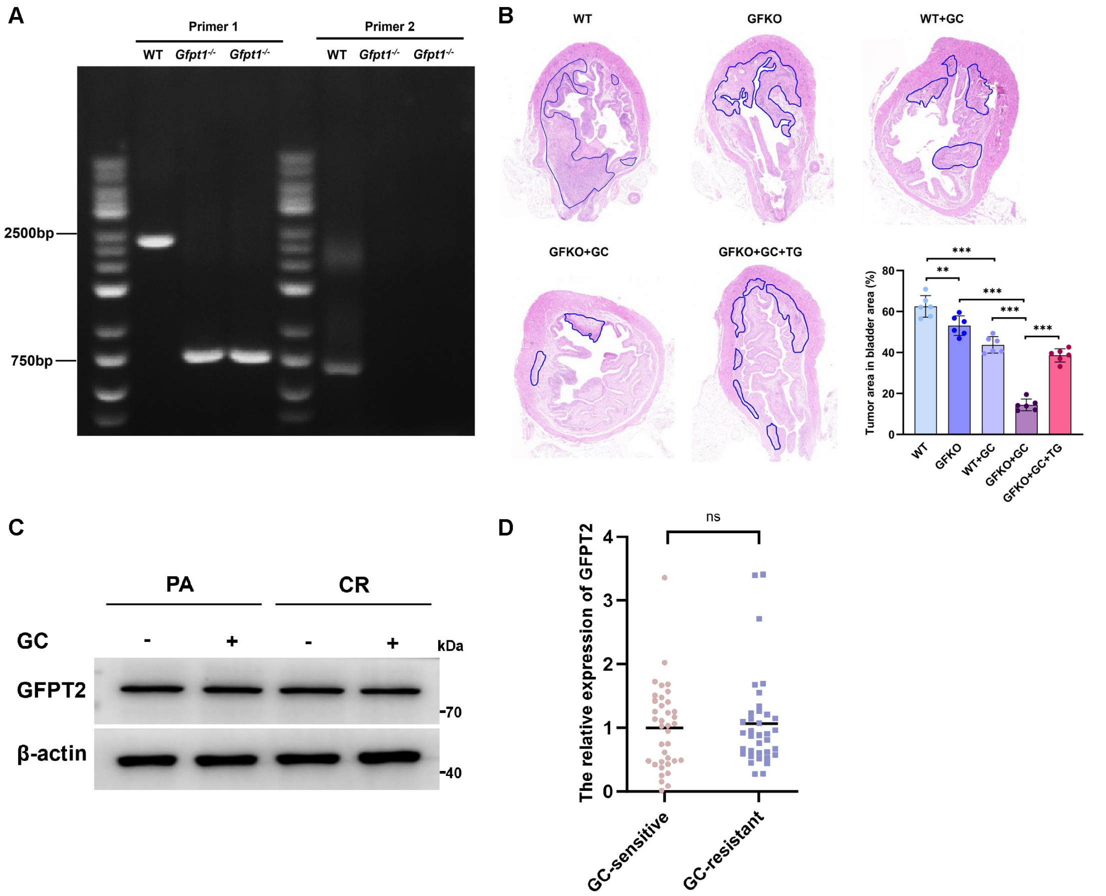

Supplementary Figure 3. Knockout of GFPT1 increased chemosensitivity in the orthotopic BCa models. A. DNA gel confirming the loss of GFPT1 gene by PCR; Primer 1 includes ACTAGGTTCAGCGGGTTTGG (F) and ACGTGCATTTAGGCAGGACA (R), Primer 2 includes ACTTGCCAGAGAAGCGTGAA (F) and GGATTCCCCTCCCAACACTG (R). **B.** Distorted blue circulars representing the bladder tumor areas in the HE-stain images, diagnosed by an experienced pathologist; the tumor burden is quantified by using the percentage of tumor area in the whole bladder area (n = 6/group). **C.** The protein levels of GFPT2 in PA and CR cell lines treated with GC (IC50) or not. **D.** The relative GFPT2 transcriptional expression in post-chemotherapy tumor tissues from GC-sensitive (n = 37) and GC-resistant (n = 36) BCa patients; normalized according to the GFPT1 levels of GC-sensitive BCa tissues; unpaired two-tailed Student's t-test. ***$p < 0.001$ represents a significant difference between two groups; ns represents no significant difference.

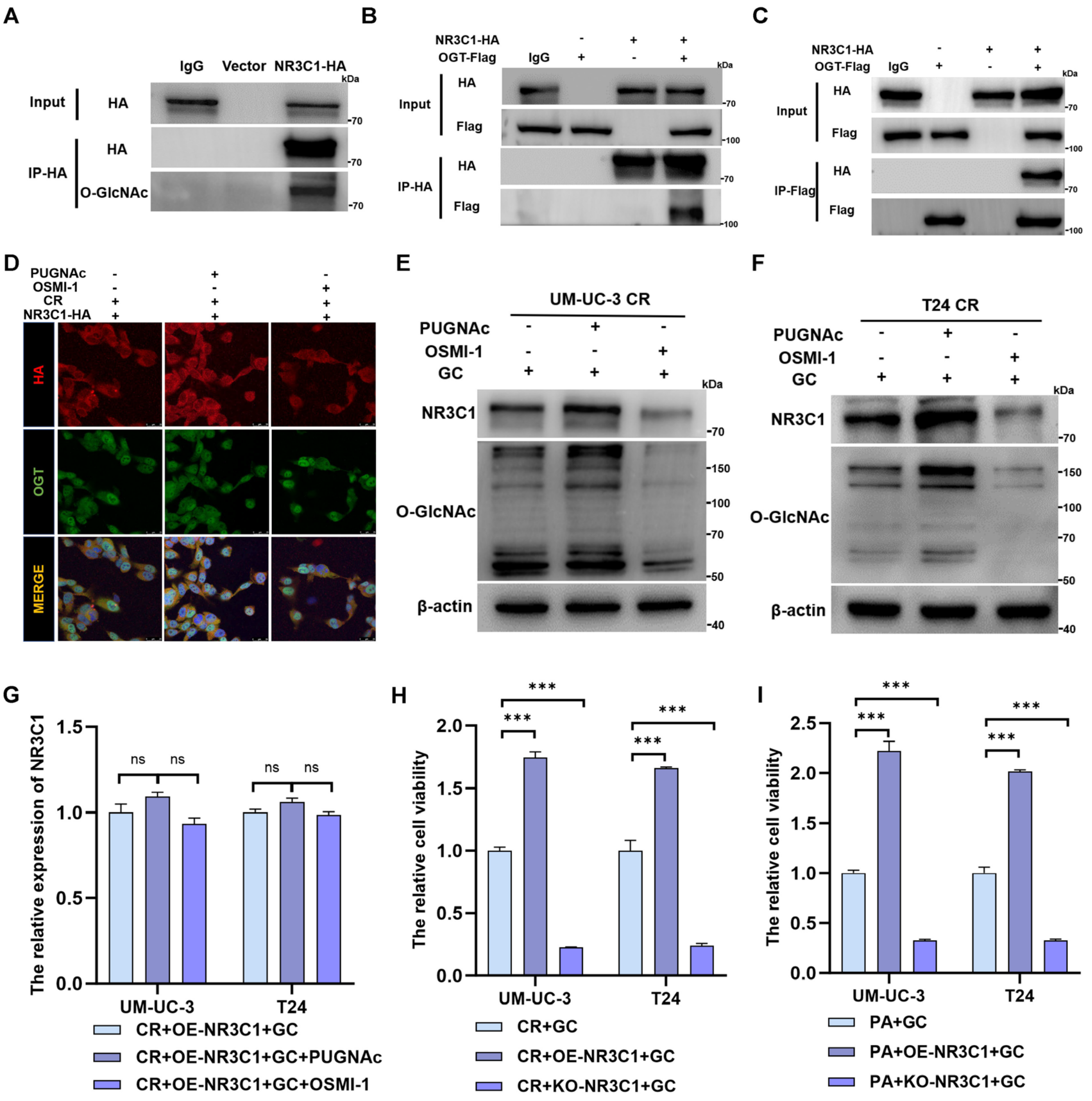

Supplementary Figure 4. O-GlcNAcylation increases the translational level of GR and decreases the sensitivity to GC in BCa. A. IP assays using an anti-HA antibody in CR cells transfected with GR-HA or a vector control; WB assays were detected by using anti-O-GlcNAc and anti-HA antibodies. Co-IP assays of GR-HA and OGT-Flag were identified by an anti-HA antibody (**B**) or an anti-Flag antibody (**C**). **D.** The IF assays exhibited the subcellular localization and co-localization of GR-HA (Red) and OGT (Green) following the administration with 50 µM PUGNAc or 50 µM OSMI-1 for 24 h. The translational (**E-F**) and transcriptional (**G**) levels of GR in CR cells treated with 50 µM PUGNAc, 50 µM OSMI-1 or not; normalized according to the GR level in CR+OE-GR+GC; one-way ANOVA followed by Tukey's test (n = 4/group). **H-I.** CCK-8 assays showed cell viabilities in GC-treated CR and PA cell lines with different GR levels; normalized according to the cell viabilities of CR+GC or PA+GC; one-way ANOVA followed by Tukey's test (n=4/group). ***$p < 0.001$ represents a significant difference between two groups; ns represents no significant differences; ns represents no significant difference.

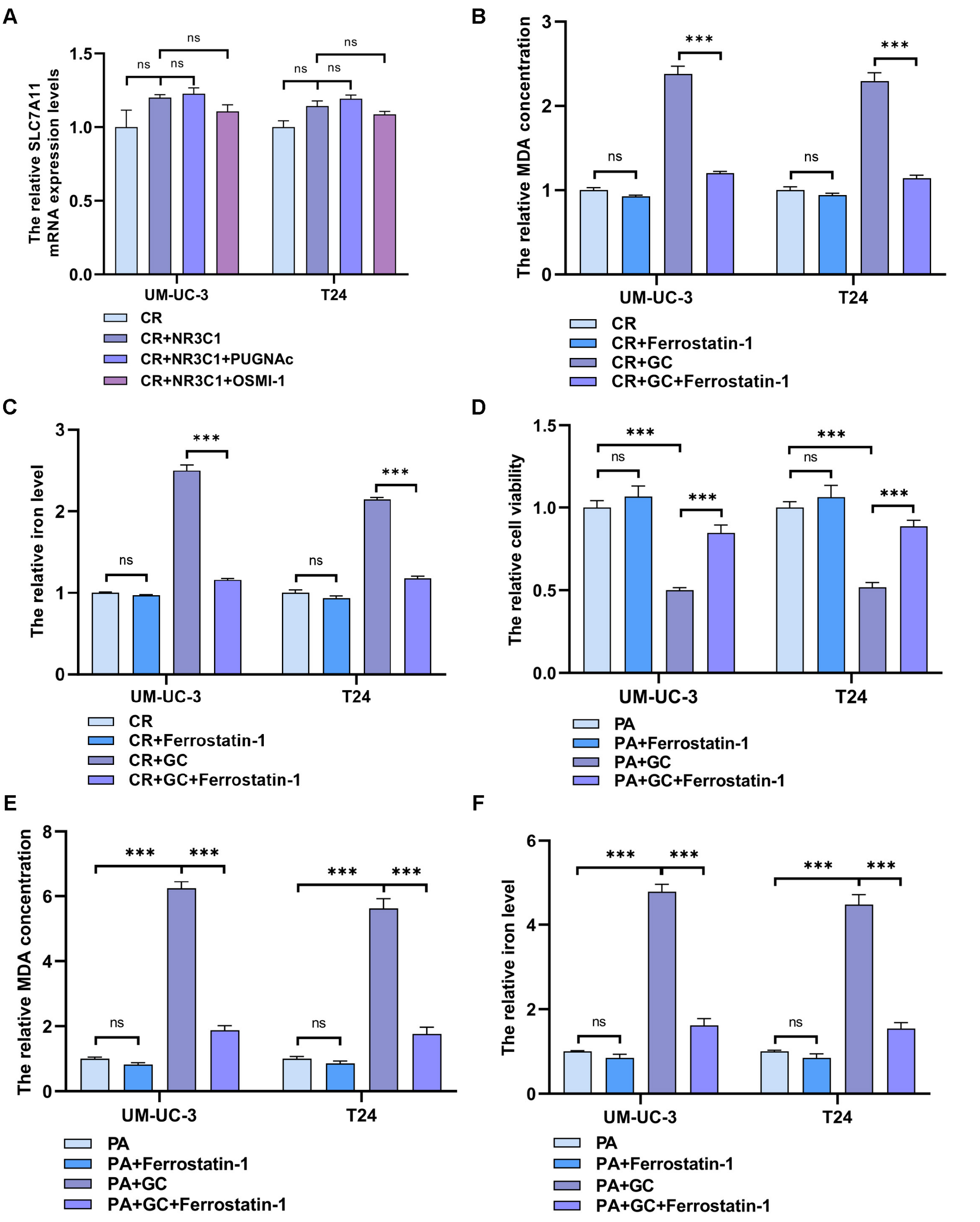

Supplementary Figure 5. Inhibition of ferroptosis decreases the sensitivity to GC in BCa. A. The relative SLC7A11 mRNA expression in CR cells transfected with NR3C1 or NR3C1-T299A, following treatments with 50 μM PUGNAc or 50 μM OSMI-1 for 24 h; normalized according to the expression of CR; one-way ANOVA followed by Tukey's test (n = 4/group).The relative MDA concentration (**B**) and iron level (**C**) in CR cell lines treated with 1 μM Ferrostain-1 and (or) GC (IC50 of CR cell lines); normalized by the levels of CR; one-way ANOVA followed by Tukey's test (n = 4/group). **D.** CCK-8 assays detecting the relative cell viabilities of PA cell lines treated with 1 μM Ferrostain-1 and (or) GC (IC50 of PA cell lines); normalized by the levels of PA; one-way ANOVA followed by Tukey's test (n = 4/group). The relative MDA concentration (**E**) and iron level (**F**) in PA cell lines treated with 1 μM Ferrostain-1 and (or) GC (IC50 of PA cell lines); normalized

by the levels of PA; one-way ANOVA followed by Tukey's test (n = 4/group). ***p < 0.001 represents a significant difference between two groups; ns represents no significant differences.

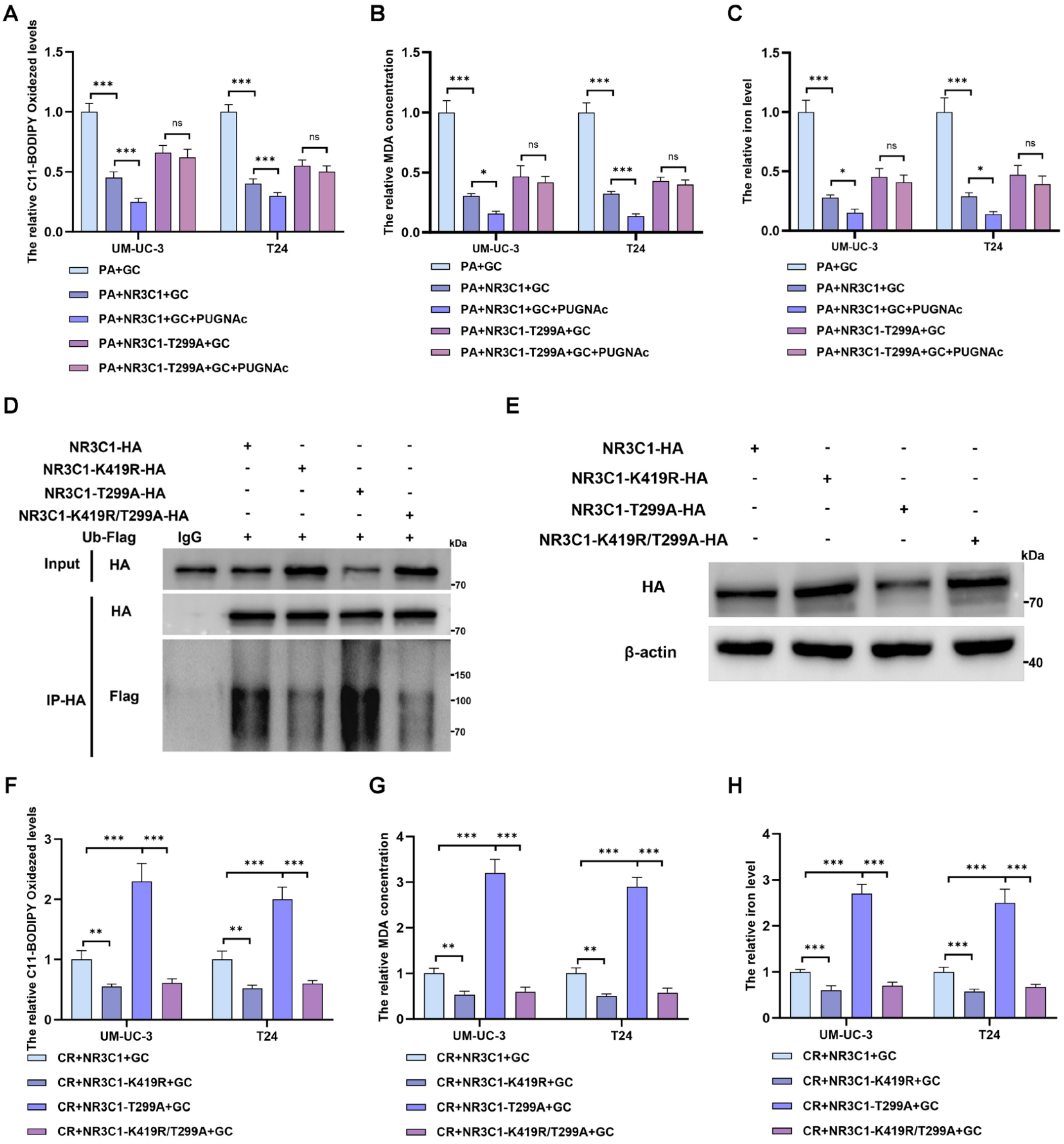

Supplementary Figure 6. NR3C1 O-GlcNAcylation at Thr^{299} reduces GC-induced ferroptosis by inhibiting its ubiquitination. Following specified gene intervention (WT or T299A mutation) of NR3C1, respectively, PA cells were treated with or without 50 μM PUGNAc for 24 h. Subsequently, the relative lipid peroxidation levels via C11-BODIPY fluorescent probe (**A**), MDA concentrations (**B**) and iron levels (**C**) were conducted; normalized according to the levels in PA+GC group; one-way ANOVA followed by Tukey's test (n=4/group). (**D**) The ubiquitination assays of HA-tagged NR3C1 wild-type and site-specific mutants in CR cells transfected with Flag-tagged ubiquitin by using an anti-HA antibody; WB assays were performed using anti-Flag and anti-HA antibodies; Flag-tagged ub was quantified and normalized according to the levels in IgG group; one-way ANOVA followed by Tukey's test (n=4/group). (**E**) The protein levels of NR3C1 in CR cell lines transfected with various NR3C1 variants. The relative lipid peroxidation levels via C11-BODIPY fluorescent probe (**F**), MDA concentrations (**G**) and iron levels (**H**) were conducted in

GC-treated CR cells transfected with various NR3C1 variants. ***$p < 0.001$, **$p < 0.01$ and *$p < 0.05$ represent significant differences between two groups; ns represents no significant differences.

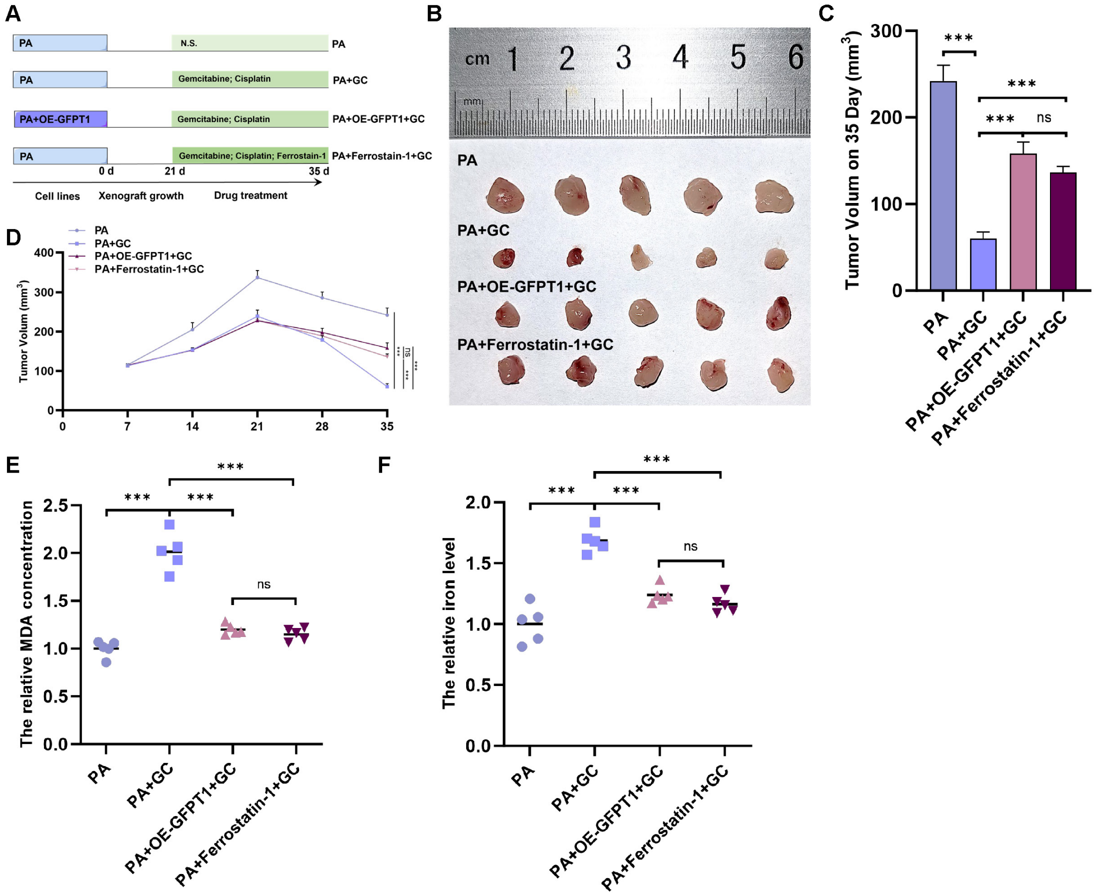

Supplementary Figure 7. GFPT1 notably decreases the sensitivity to GC by inducing ferroptosis in BCa. A. Schematic diagram of the experimental protocols. PA cells with different GFPT1 levels were subcutaneously injected into 5-week-old female BALB/c nude mice to establish PA cell line-derived xenograft mice models; normal saline, GC (gemcitabine 20 mg/kg/2d; cisplatin 2 mg/kg/2d) and (or) Ferrostain-1 (5 mg/kg/2d) were treated from the 8th week (n = 5/group). **B.** Images of PA cell line-derived xenograft tumors in 5-week-old male BALB/c nude mice (n = 5/group); GC (gemcitabine 20 mg/kg/2 d; cisplatin 2 mg/kg/2d) and (or) Ferrostain-1 (5 mg/kg/2d) were treated when tumor volumes reached 200-400 mm^3 on the 21st day after xenograft. **C.** Tumors were weighted on the 35th day after xenograft; one-way ANOVA followed by Tukey's test (n= 5/group). **D.** Tumor volumes were measured on the 7th, 14th, 21st, 28th and 35th day after xenograft; one-way ANOVA followed by Tukey's test (n= 5/group). The relative MDA concentrations (**E**) and iron levels (**F**) in xenograft tissues; normalized by the levels in PA group; one-way ANOVA followed by Tukey's test (n= 5/group). ***$p < 0.001$ represents a significant difference between two groups; ns represents no significant differences.

Table S1: The primer sequences

Name	Item	Sequence
**β-actin**	F primer	CCTTCCTGGGCATGGAGTC
	R primer	TGATCTTCATTGTGCTGGGTG
**GFPT1**	F primer	TCCCTTGTGATGTTTGCCT
	R primer	TCAGTGCCCCTTCAAGACAA
**GNA-1**	F primer	CATCCTGGAGAAGGCTTGGTT
	R primer	GCTGACAACTCCAGTCTCTGT
**PGM3**	F primer	GGCCGATATGGAAAGGCAAC
	R primer	GCCATTTGCACAGTCAACCT
**UAP**	F primer	ACCAGTGGCAGAACAATGGA
	R primer	AGTGCCCGATAAAGACCACC
**GPX4**	F primer	AAGTGGATGAAGATCCAACCCAAG
	R primer	GGGGCAGGTCCTTCTCTATCA
**NR3C1**	F primer	TGGTGTCACTGTTGGAGGTTATT
	R primer	TGCAGTAGGGTCATTTGGTCATC
**GFPT2**	F primer	GACCATCGCCAAGCTGATTAAAT
	R primer	AATGCACCTTCCAACTGCTGAAT
**Primer 1**	F primer	ACTAGGTTCAGCGGGTTTGG
	R primer	ACGTGCATTTAGGCAGGACA
**Primer 2**	F primer	ACTTGCCAGAGAAGCGTGAA
	R primer	GGATTCCCCTCCCAACACTG

Note: F primer: forward primer; R primer: reverse primer.

Table S2: The promoter sequences of GPX4

Name	Sequence (5'-3')
**GPX4-WT**	…CCCCCAAGTCAACCCGGCCCTGAGTCTGTCTCTGAAGAAATACTCAGTGCAGGGT CAGAAAAGGTTAAGACCTTGAGGACAACATACAAATCCCAAGCACCTAGGCGGGGT AGAGTGGCTCACACCTGTAATCCCAGCACTTTGGGAGGCCGAGGCAGGCGGATCAC CTGAGGTCGGGAGTTCGAGACCAGCCTGACCAACATGGTGAAAGCCCGTATCTACT AAAAACACAAAAATTAGCTGGGCGTGGTAGCACATGCCTGTAATCCCAGCTACTCA GGAGACTGAGGCAGGAGAATCACTTGAACCCGGAAAGCGGAGGTTGCAGTGAGCTG CGATGGAGCCACTGC…
**GPX4-MUT**	…CCCCCAAGTCAACCCGGCCCTGAGTCTGTCTCTGAAGAAATACTCAGTGCAGGGT CAGAAAAGGTTAAGACCTTGAGGACAACATACAAATCCCAAGCACCTAGGCGGGGT AGAGTGGCTCACACCTGTAATCCCAGCACTTTGGGAGGCCGAGGCAGGCGGATCAC CTGAGGTCGGGAGTTCAGAGTTGATTCAGTTGGTATGGTGAAAGCCCGTATCTACT AAAAACACAAAAATTAGCTGGGCGTGGTAGCACATGCCTGTAATCCCAGCTACTCA GGAGACTGAGGCAGGAGAATCACTTGAACCCGGAAAGCGGAGGTTGCAGTGAGCTG CGATGGAGCCACTGC…

Note: The mutant of binding site is marked red. WT: wide-type; MUT: mutant.
